# The incidence of acute encephalitis syndrome in Western industrialised and tropical countries

**DOI:** 10.1186/1743-422X-5-134

**Published:** 2008-10-30

**Authors:** Fidan Jmor, Hedley CA Emsley, Marc Fischer, Tom Solomon, Penny Lewthwaite

**Affiliations:** 1 Division of Neuroscience, University of Liverpool, Clinical Sciences Centre, Lower Lane, Liverpool, L9 7LJ, UK; 2 Brain Infections Group, Divisions of Neuroscience and Medical Microbiology, School of Tropical Medicine, University of Liverpool, Liverpool, L9 7LJ, UK; 3 Centers for Disease Control and Prevention, Atlanta, Georgia, USA

## Abstract

**Background:**

As part of efforts to control Japanese encephalitis (JE), the World Health Organization is producing a set of standards for JE surveillance, which require the identification of patients with acute encephalitis syndrome (AES). This review aims to provide information to determine what minimum annual incidence of AES should be reported to show that the surveillance programme is active.

**Methods:**

A total of 12,436 articles were retrieved from 3 databases; these were screened by title search and duplicates removed to give 1,083 papers which were screened by abstract (or full paper if no abstract available) to give 87 papers. These 87 were reviewed and 25 papers identified which met the inclusion criteria.

**Results:**

Case definitions and diagnostic criteria, aetiologies, study types and reliability varied among the studies reviewed. Amongst prospective studies reviewed from Western industrialised settings, the range of incidences of AES one can expect was 10.5–13.8 per 100,000 for children. For adults only, the minimum incidence from the most robust prospective study from a Western setting gave an incidence of 2.2 per 100,000. The incidence from the two prospective studies for all age groups was 6.34 and 7.4 per 100,000 from a tropical and a Western setting, respectively. However, both studies included arboviral encephalitis, which may have given higher rather than given higher] incidence levels.

**Conclusion:**

In the most robust, prospective studies conducted in Western industrialised countries, a minimum incidence of 10.5 per 100,000 AES cases was reported for children and 2.2 per 100,000 for adults. The minimum incidence for all ages was 6.34 per 100,000 from a tropical setting. On this basis, for ease of use in protocols and for future WHO surveillance standards, a minimum incidence of 10 per 100,000 AES cases is suggested as an appropriate target for studies of children alone and 2 per 100,000 for adults and 6 per 100,000 for all age groups.

## Background

As part of the effort to control Japanese encephalitis (JE), the World Health Organization (WHO) is producing a set of standards for JE surveillance [1]. The surveillance consists of identifying patients with acute encephalitis syndrome (AES), and then classifying the patients according to the results of laboratory diagnostic tests. AES is defined as the acute onset of fever and a change in mental status (including symptoms such as confusion, disorientation, coma, or inability to talk) and/or new onset of seizures (excluding simple febrile seizures) in a person of any age at any time of year. As with all surveillance standards, the document includes performance targets that give an indication of the quality of the surveillance. The minimum annual incidence of a disease syndrome that one would expect to be reported provides a vital indication of whether surveillance is active. For example in the polio eradication surveillance standards, an annual rate of non-polio acute flaccid paralysis cases of 1 per 100,000 children is the minimum that should be reported to show that surveillance is active [2]. A performance target for the minimum annual incidence of AES was not defined in the field test version of the Japanese encephalitis surveillance standards [1], pending further information about the likely minimum incidence of AES.

This review provides information to answer the question: What is the minimum annual incidence of AES that should be reported per 100,000 population to show that the surveillance programme is active? Although there are no studies that specifically address the incidence of AES (a broad syndromic definition that includes many patients who do not have encephalitis [3]), there are studies looking at the incidence of encephalitis in different settings. The surveillance standards have been devised for JE control and it is envisaged that in JE endemic areas, the number of cases will fall as the disease control programmes are further implemented. Thus in addition to looking at the incidence of JE in areas currently or historically endemic for JE we examined the incidence of AES in Western industrialised areas where JE does not occur.

## Results

A total of 12,436 articles were retrieved from 3 databases; these were screened by title search and duplicates removed to give 1,083 papers which were screened by abstract (or full paper if no abstract available) to give 87 papers (Table 1). These 87 were reviewed and 25 papers identified which met the inclusion criteria. All relevant studies for producing the recommended incidence to be used in the WHO surveillance standards included are described below. Initially, 87 articles were considered [see Additional file 1] and 25 articles were finally chosen because they met the selection criteria and were representative of the spectrum of study type and disease incidence (Tables 2 and 3). Studies were evaluated and reviewed to determine the incidence of AES, specific infectious aetiologies of encephalitis in various age groups and geographic areas, and case definitions employed.

**Table 1:** Literature search strategy and results.

Search	Search Strategy	Dates of original search	Numbers of articles (No limits)	Title screened articles	Totals from title screen	Duplicates removed	Further Title & Abstract or paper screen
1 Pubmed	"Incidence or epidemiology" AND "encephalitis".	1950-autumn 2007	6895	479		1083	87
			
2 Pubmed	"encephalitis and epidemiology" AND" Japanese encephalitis Virus and Incidence"	1950-autumn 2007	492	66 (-34 duplicates) = 32	527		
			
3 Pubmed	"encephalitis and epidemiology" AND "herpes and Incidence"	1950-autumn 2007	266	43(-27 duplicates) = 16			
			
4 OVID	"Incidence or epidemiology" AND "encephalitis".	1950-autumn 2007	Exploding each term 509 1467 – 48 duplicates = 1419	445	445		
			
5 EMBASE	"Incidence or epidemiology" AND "encephalitis". [Limits English, Human]	1966-autumn 2007	1631 exploded terms 3369 – duplicates 3364	300	300		

Totals			12,436	1,272	1,272	1,083	87

**Table 2:** Aetiology and outcome for each of the selected AES studies*

Study (Publication Year)	Study Year	Ages	Aetiology confirmed	Fatality Rate%	Sequelae %
Klemola et al. (1965)[5], Kaeaeriaeinen et al. (1964)[6]	1945–1963	All ages	40% from 1958 onwards	10	33

Beghi et al. (1984)[4]	1950–1981	All ages	15.3	3.8	not reported

Henrich et al. (2003).[35]	1993–1998	All ages	not reported	not reported	not reported

Pedersen (1956)[37]	1952–54	All ages	not reported	not reported	not reported

Nicolosi(1986)[36]	1950–1981	All ages	15	3.8	not reported

Ponka et al. (1982)[13]	1980	All ages	not reported	not reported	not reported

Khetsuriani et al. (2002)[18]	1988–1997	All ages	40.5	7.4	not reported

Khetsuriani et al. (2007)[34]	1988–1997	All ages	81.5	100	not applicable

Kamei et al. (2000)[17]	1989–1991	All ages	48.8	not reported	not reported

Davison et al. (2003)[20]	1989–1998	All ages	40.1	9.7	56 (mild deficits)

Trevejo (2004)[19]	1990–1999	All ages	43.7	not reported	not reported

Mailles, et al. (2007)[21]	2000–2002	All ages	16.8	15–28	not reported

Rantalaiho et al. (2001)[7]	1967–1991	>= 15 yrs	50.6	5.6	not reported

Radhakrishnan et al. (1987)[38]	1983–1984	>15 yrs	0	20	not reported

Nwosu et al.(2001)[33]	1991–1993	>= 16 yrs	0	50	0

Kupila et al. (2006)[9]	1999–2003	>= 16 yrs	35.7	not reported	71 (mild-severe deficits)

Koskiniemi et al. (1991) [8]	1968–1987	1 mths-16 yrs	68	3	7 severely damaged

Rantakallio et al. (1986)[39]	1966–1972	<14 yrs	38.1	not reported	not reported

Koskiniemi et al. (1997)[11]	1983–1984	1 mths-15 yrs	62.9	not reported	not reported

Ilias et al. (2006)[14]	2000–2004	<14 yrs	72.2	0	not reported

Wang et al. (1981)[40]	1972–1980	<=16 yrs	36 of all viral CNS infections	not reported	not reported

Rantala & Uhari (1989)[12]	1973–1987	<16 yrs	61.1	1.1	not reported

Wong et al. (1987)[41]	1975–1986	<14 yrs	26	28	24

Cizman et al. (1993)[15]	1979–1991	1mths-15 yrs	68.2	1.8	24 (neurological deficits)

Ishikawaet al. (1993)[16]	1984–1990	<15 yrs	41	7.8	66 (neurological deficits)

*Studies covering all ages are listed first, followed by adult studies and then paediatric studies. Within each section the studies are ordered by type; longitudinal (L), then prospective (P) and then retrospective (R) studies. Yrs Years, Mths Months.

**Table 3:** Summary of AES incidence rates in the selected studies

**Study (Publication Year)**	**Year of Study**	**Setting Country Western (W)/Tropical (T)**	**Study Design Longitudinal (L) Prospective (P)/Retrospective (R)/**	**Ages**	**Incidence per 100,000 population**
Klemola et al (1965), Kaeaeriaeinen et al(1964)[5,6]	1945–1963	Finland (W)	L	All ages	2 to 3

Beghi et al. (1984)[4]	1950–1981	USA (W)	P	All ages	7.4

Henrich et al. (2003)[35]	1993–1998	Thailand (T)	P	All ages	6.34

Pedersen (1956)[37]	1952–54	Jutland (W)	R	All ages	6.75–9.25**

Nicolosi (1986)[36]	1950–1981	USA (W)	R	All ages	7.4

Ponka et al. (1982)[13]	1980	Finland (W)	R	All ages	3.5

Khetsuriani et al. 2002)[18]	1988 – 1997	USA (W)	R	All ages	7.3

Khetsuriani et al. (2007)[34]	1988–1997	USA (W)	R	All ages	0.51–0.53*(deaths)

Kamei et al. (2000)[17]	1989–1991	Japan (T)	R	All ages	1.77 *(± 0.32)

Davison et al. (2003)[20]	1989–1998	England (W)	R	All ages (children <17 yrs)	1.5 (2.8 in children) (1.1 in adults)

Trevejo (2004)[19]	1990–1999	USA (W)	R	All ages	4.3 (CI 4.2–4.4)

Mailles et al. 2007)[21]	2000–2002	France (W)	R	All ages	1.9

Rantalaiho et al. (2001)[7]	1967–1991	Finland (W)	L	Adults ≥ 15 yrs	1.4

Radhakrishnan et al. (1987)[38]	1983–1984	Libya (T)	P	Adults >15 yrs	1

Nwosu et al. (2001)[33]	1991–1993	Nigeria (T)	P	Adults ≥ 16 yrs	0.9

Kupila et al. (2006)[9]	1999–2003	Finland (W)	P	Adults ≥ 16 yrs	2.2

Koskiniemi et al. (1991)[8,10]	1968–1987	Finland (W)	L	Children 1 mths-6 yrs	8.3 (range 19.8 in 1974 to 2.5 in 1986 and 1987)

Rantakallio et al. (1986)	1966–1972	Finland (W)	P	Children <14 yrs (1966 birth cohort)	12.6

Koskiniemi et al. (1997)[11]	1993–1994	Finland (W)	P	Children 1 mths-15 yrs	10.5

Ilias et al. (2006)[14]	2000–2004	Greece (W)	P	Children <14 yrs	13.8**

Wang et al. (1981)[40]	1972–1980	Canada (W)	R	Children ≤ 16 yrs	8.2**

Rantala & Uhari (1989)[12]	1973–1987	Finland (W)	R	Children <16 yrs	8.8

Wong et al. (1987)[41]	1975–1986	Hong Kong (T)	R	Children <14 yrs	14.25**

Cizman et al. (1993)[15]	1979–1991	Slovenia (W)	R	Children 1 mths-15 yrs	6.7 (range 2.37–12.6)

Ishikawa et al. (1993)[16]	1984–1990	Japan (T)	R	Children <15 yrs	3.3

* Incidence converted to/100,000, ** incidence calculated from data in paper. Yrs Years. Mths Months, P prospective, R retrospective, L Longitudinal, CI confidence interval. Studies covering all ages are listed first followed by adult studies and then paediatric studies. Within each section the studies are ordered by type; longitudinal, then prospective and then retrospective studies.

For the purpose of this paper, articles assessing AES were categorised into Western industrialised and tropical settings, where Western countries were defined as belonging to Europe and North America. Nineteen articles discussed AES in Western industrialised countries and the remaining 6 looked at AES in Tropical countries (Tables 2 and 3) and [see Additional file 1]. For the 62 additional papers from Western countries where specific disease incidences were given, viral agents included St Louis encephalitis virus (SLEV), tick-borne encephalitis virus (TBEV), herpes simplex virus-1 and 2 (HSV-1 &2), measles virus, West Nile virus (WNV) and varicella zoster virus (VZV) [see Additional file 1]. These studies illustrate that arboviruses are also important causes of encephalitis in Western settings but there is a greater range of viruses reported than in tropical settings, many of which like JE are vaccine preventable. From tropical countries Japanese encephalitis virus (JEV) studies predominated.

### Incidence of encephalitis of any cause

Of the 25 studies that met the selection criteria, fourteen are discussed here in more detail as they include the studies from which the incidences for surveillance data are suggested, as well as examples of other similar studies from Western and tropical settings. More information is available from these and all 87 papers [see Additional file 1].

In a prospective population-based study, all cases fulfilling a case definition for encephalitis were identified for the period 1950–1981, in Olmsted County, Minnesota, USA [4]. Cases considered as 'possible' were excluded from all calculations, even though age distribution, seasonal trends and residence were similar to 'confirmed' cases. Although all causes were studied, mumps virus and the California serogroup viruses were the most commonly identified causes and by the last decade of the study, the rate at which a viral aetiology was determined had improved to approximately 25%. The overall annual incidence of 7.4 per 100,000 remained stable over successive 5 to 10 year periods. The case fatality rate was 3.8%.

There have been a large number of studies of encephalitis from Finland, the earliest of these is a longitudinal study from 1945–1963 [5,6] in which patients of all ages with an acute infectious disease of apparent viral aetiology were studied. Virological diagnostics changed over the course of the study and viral isolation was introduced as routine in 1952. In this study the overall incidence of viral encephalitis was found to be 2 to 3 per 100,000.

A more recent longitudinal hospital-based study covering the period 1967 to 1991 aimed to provide good surveillance coverage of acute encephalitis over a large part of Southern Finland [7]. Of 322 adult patients, aetiology was confirmed, probable or suggested in 51%, with HSV being the most frequent causative agent. Employing stringent exclusion, case definition and laboratory diagnostic criteria, an annual incidence of 1.4 per 100,000 was reported. Mumps virus was the leading cause of acute encephalitis until effective vaccination programmes virtually eliminated it from Finland [8].

Patients aged 16 or over admitted to one main hospital in Finland between 1999 and 2003 were included in a study investigating encephalitis and aseptic meningitis [9]. This prospective study provided detailed case and laboratory diagnostic criteria and aimed to investigate the effectiveness of polymerase chain reaction (PCR) in the diagnosis of encephalitis. Aetiology was defined in 36% of patients after microbiological testing, with VZV, HSV and TBEV the major causative agents. An annual incidence of 2.2 per 100,000 encephalitis cases was reported.

A study of 462 children with encephalitis, aged 1 month to 16 years was conducted between 1968 and 1987 in Helsinki, Finland and hospital admission data for the 462 children were collated [8]. The average incidence of encephalitis was 8.8 per 100,000 (range 19.8 in 1974 to 2.5 in 1985 and 1986). Further data from this cohort showed that highest incidence of encephalitis was in children under 1 year of age (annual incidence 16.7 per 100,000); the incidence remained quite high until 10 years of age, and then gradually declined for children up to the age of 15 years (annual incidence 1.0 per 100,000) [10]. Since 1983, when measles, mumps and rubella (MMR) vaccine was introduced, the major associated agents were *Mycoplasma pneumoniae*, enteroviruses and adenoviruses. Morbidity and mortality rates were not specified.

A prospective multicentre study in Finland identified 175 cases of acute encephalitis in children aged 1 month to 15 years, and reported an overall annual incidence of 10.5 per 100,000 [11]. Again, the highest annual incidence occurred in the youngest children (18.4 per 100,000 in children under 1 year of age). In this later study, which followed the virtual elimination of encephalitis due to measles, mumps or rubella viruses, the microbial diagnosis was proven or suggested in 110 cases (63%), with VZV, respiratory or enteroviruses comprising 61% of these, and adenovirus, Epstein Barr virus, HSV and rotaviruses comprising another 5% each.

A retrospective population-based study of children with encephalitis was conducted between 1973 and 1987 in an area of Finland where there were no arboviral infections [12]. Ninety-five children were identified, giving an annual incidence of 8.8 per 100,000. The most commonly identified agents, based on virological and serological studies were VZV (24 cases), mumps virus (8), HSV (7) and measles virus (4). The aetiology remained unknown in 37 children (39%). No cases of encephalitis caused by mumps virus, measles virus or rubella virus were found in the population after 1982, when the MMR vaccine against these viruses was introduced. This was also noted by other researchers in Finland [8].

In a small prospective study of central nervous system (CNS) infections in Helsinki, Finland in 1980, 146 patients were diagnosed with CNS infections with a cerebrospinal fluid (CSF) pleocytosis (>10 cells/mm^3^), of whom 9 had meningo-encephalitis or encephalitis. This prospective data together with retrospectively collected data for the same period from other hospitals in Helsinki gave an incidence hospitals in of 3.5 per 100,000 [13].

A retrospective study performed in Heraklion, Crete from 2000 to 2004, identified 18 children hospitalised for acute encephalitis, giving an incidence rate of 13.8 per 100,000 [14]. Aetiology was attributed to viral causes in 8 cases, bacterial in a further 5 and a remaining 5 cases were of unknown cause. The absence of measles, mumps or rubella virus associated encephalitis cases was interpreted by the authors to be consistent with the elimination of these encephalitides in the post vaccine era. Although no fatalities occurred, 5 children presented at a median of 4 years after their initial infection with mild to moderate sequelae.

In Slovenia, a retrospective study covering a 13 year period from 1979 to 1991 identified 170 children (aged 1 month to 15 years) with encephalitis, giving an incidence of 6.7 per 100,000 [15]. A definite or probable aetiology was determined in 68% of cases, with TBEV (28.8%), VZV (17.0%), HSV (10.0%) being the most common agents. A subgroup of 42 children with encephalitis and focal neurological signs was identified, among whom the most common confirmed or presumed infective agent was HSV (40.4%).

A questionnaire-based epidemiological study in Japan covering a 7-year period from 1984 to 1990 identified 256 patients with acute encephalitis [16]. The authors estimated an overall annual incidence of encephalitis of 1.77 (+/-3.2) per 100,000 in children [17]. Among 105 aetiologically diagnosed cases, the most common causative agents were measles virus (23%), rubella virus (23%) and HSV (20%). The short term outcome was death in 20 cases and varying degrees of neurological sequelae including subsequent epilepsy, visual and auditory deficits and mental/physical impairment in a further 24%.

An analysis of United States National Hospital Discharge Survey data from the period 1988 to 1997 revealed an average encephalitis-associated hospitalisation rate of 7.3 per 100,000 [18]. Herpetic and toxoplasmic encephalitides were the most common individual diagnoses with known agents. Encephalitis-associated hospitalisation rates were highest for children less than 1 year of age and persons aged 65 years or over. A fatality rate of 7.4% was reported. Another analysis of US hospital discharge data for the period 1990 to 1999 reported an overall incidence rate of 4.3 per 100,000 for encephalitis admissions in California [19]. Once again the highest rate was found in infants aged less than 1 year.

In a retrospective analysis of hospitalisations for a diagnosis of viral encephalitis in England between 1989 and 1998, 6414 cases were identified [20]. Most cases (60%) were of unknown aetiology, but HSV was the most frequent causative agent among cases with an identified cause, accounting for 52%. An overall annual incidence rate of 1.5 per 100,000 was reported. The highest rate (8.7 per 100,000) was for children under 1 year of age. The overall case-fatality rate was 6.5%. Significant under-reporting of viral encephalitis was noted when routine systems were compared to the hospital episode statistics, with 97% of hospitalised cases not being formally reported.

Most recently, a retrospective French population-based study undertaken between 2000 and 2002, using the national hospital medical database, found an annual average incidence of acute encephalitis of 1.9 per 100,000 [21]. Aetiological diagnosis was not made in 80% of cases, but HSV and VZV were the most common identified causes. Comparison with other studies was hampered by the exclusion of patients infected with HIV. The fatality rate was 6% and by 6 months, 71% patients experienced moderate to severe sequelae.

### Incidence of Japanese encephalitis

Many studies set in Western industrialised countries tend to focus on HSV, TBEV, SLEV, WNV or bacterial encephalitides [see Additional file 1]. In contrast, tropical countries are faced with high incidences of JE and so the majority of studies arising from Southeast Asia, Japan and China, for example, are focused on the epidemiology of JE.

Tigertt *et al*. (1957) conducted a programme in Japan over a 4-year period before 1950 aiming to investigate the efficacy of a formalin-inactivated mouse-brain vaccine in an area where JE was relatively common [22]. Children recruited from Okayama Prefecture in Japan were administered 3 doses of vaccine. Although no cases of AES were reported to have occurred in 1946, in the subsequent 4 years there was an overall rate of 41.2 per 100,000 across the region. However, a dramatically lower rate of 11.8 per 100,000 was observed in those vaccinated under the study programme.

A prospective study set in Chiangmai Valley, Thailand in 1970 reported the annual incidence among residents of the valley to be 14.7 per 100,000 [23]. The area had known geographic boundaries and a population that could be described demographically with reasonable accuracy thus allowing the cases themselves to help describe the temporal and spatial occurrence of JE. A similarly high incidence has been reported for other epidemic areas of Southeast Asia such as in Northern Vietnam where between 1969 and 1974, incidence rates of 8.7 to 22.0 per 100,000 were reported [24].

Between 1968 and 1971 JE surveillance in Taiwan was conducted with WHO-assisted programmes. Okuno *et al*. (1975) found a clear pattern of JE consistently occurring in mid July mostly in the southernmost county of Taiwan [25,26]. The study population was that of Taiwan itself and other offshore islands such as the Pescadores. After patients were identified, blood and convalescent sera were obtained, and of 1,024 patients, 273 were confirmed as JE. An incidence of between 2 and 7 per 100,000 was reported across the study period. Case fatality never exceeded 25%.

Unlike the other studies carried out in tropical settings, Hoke *et al*. (1988) performed a placebo-controlled, blinded randomised clinical trial in a northern Thai Province between 1984 and 1985 to assess the efficacy of 2 different vaccines [27]. A cohort of children aged between 1 and 14 years was selected from 12 schools for the study. A marked difference in incidence of JE was observed in the non-vaccinated compared to the vaccinated groups; 51 per 100,000 compared to 5 per 100,000 respectively.

A wide range of incidence of JE in Thailand was noted during the late 1980s, with the northern provinces particularly affected with average incidences of 5 per 100,000 compared to other provinces with an average incidence of 2 per 100,000 (range 1.69–6.68 per 100,000). JE was noted to be less common in southern and central areas than in the north [28]. The majority of cases (85%) were in those under 25 years of age and 66% of all cases occurred in children less than 15 years of age, with the highest rate in those aged 5 to 9 years. No specific detail for case definition or laboratory diagnosis was provided, although the overall conclusions of this study seem to agree with other detailed studies set in the same location [25,29]. The study highlights the importance of good epidemiological data to appropriately target JE control measures.

In Taiwan the incidence of JE is reported to have decreased since the introduction of vaccination in 1968. This is evident from a prospective population-based study carried out here over a 30-year period where the annual incidence of 2.05 per 100,000 in 1967 dropped to 0.03 per 100,000 in 1997 [30]. This study excluded non-confirmed cases and non-JE cases. The dramatic decrease in JE occurrence was attributed to a combination of the improved living conditions due to urbanisation, and use of insecticides coupled with a lasting childhood immunisation campaign. Although the mortality rate decreased in line with incidence, 40% of all patients who survived JE developed sequelae.

More recently in Bali, a prospective hospital-based surveillance study of children younger than 12 years gave an incidence of JE of 7.1 per 100,000 from a population of approximately 600,000 where 86 confirmed and 4 probable cases were identified [31]. The incidence was notably higher in the southern plains than in the mountainous districts of northern Bali, and was attributed to a wider distribution of rice fields in the south. Among survivors 37% had neurological sequelae at discharge and 10% of the children died. In epidemic years the incidence of JE can be much higher than these, with incidences as high as 185 reported from an outbreak in Nepal in 1997 [32]. These findings contradict suggestions that JE is rare in tropical Asia and suggest the geographical range of JE is broader than previously thought. Vaccination implementation is being considered in Bali.

## Discussion

This review has shown a wide range of reported incidences for acute encephalitis, both in tropical and Western industrialised settings, ranging from 0.9 per 100,000 for adults in Nigeria [33], to 185 per 100,000 for a rural population during a JE outbreak in Nepal [32]. The key question we set out to address was what is the minimum incidence of acute encephalitis syndrome that should be reported to show that there is active surveillance taking place? We have found that in the most robust prospective studies conducted in Western industrialised countries, a minimum incidence of 10.5 per 100,000 AES cases was reported for children and 2.2 per 100,000 for adults. A minimum incidence rate of 6.34 per 100,000 was reported for all ages from a tropical setting.

To reach this answer, we looked at the incidence of encephalitis in a range of studies. Our analysis is hindered to some extent by the very different methodologies used. In addition it should be remembered that the number of patients with a final diagnosis of encephalitis is likely to be considerably lower than the number that meet the rather broad WHO case definition of acute encephalitis syndrome on hospital admission (in essence any patient with a febrile illness, altered consciousness and/or seizures). For example, a recent assessment of the WHO JE case definitions in Vietnam has shown that only 88 (30%) of 296 patients with acute encephalitis syndrome had a final diagnosis of encephalitis; the most common final diagnoses were meningitis, other infectious encephalopathies, and non-infectious causes [3].

### Case definitions and diagnostic criteria

Of the 12 studies which looked at incidence of AES in all age groups, 5 studies relied on WHO International Classification of Disease (ICD) codes (9^th ^edition [18,19,34], 10^th ^edition [21], or both [20]) in place of clinical case definitions for cases of suspected acute encephalitis. Although ICD codes used on discharge provide a useful means of comparing data between hospitals [18] and studies comparing different populations, they can hinder precise aetiological diagnosis due to their generic nature. Additionally, because the unit of analysis is a hospital discharge and not a patient, multiple hospital admissions by the same patient can lead to difficulties in determining the true incidence rate in a given study [18]. One study attempted to overcome this problem by recording the record linkage number (relating to a patient's social security number) wherever possible, to identify patients with multiple hospitalisations [19].

Case definitions for encephalitis, where specified, varied remarkably between studies and made comparisons difficult. Of the 25 studies of AES, apart from the 5 studies relying on WHO ICD codes, 15 [4,5,7,13-17,35-41] gave no detailed clinical case definition for an acute encephalitis case, including two studies in which diagnosis was at the discretion of the consulting physician [16,37]. Considerable variation was noted in case definitions employed by the remaining 5 studies [9-12,33]. Use of standardised case definitions will be important for the reliability of data from future studies. Three studies failed to provide exclusion criteria [13,20,35].

### Aetiology confirmation

No laboratory diagnostic criteria were specified in 10 studies [5,16-18,33,34,37-40]. Most studies that specified laboratory diagnostic criteria relied on a combination of complement fixation and haemagglutination inhibition in CSF and/or serum, using either single or paired specimens from different phases of each patient's infection. Evaluating antibody titres may be problematic however, especially in young children as development of antibody, particularly in CSF may occur late [11]. Serum antibodies may indicate an associated infection rather than a causative CNS infection and could potentially lead to a falsely high incidence rate if not taken into account.

Despite extensive microbiological investigation, there was always a significant proportion of patients with no causative agent identified (28–85% unconfirmed). Two principle explanations for this might be firstly that the screening techniques used were not adequate to detect all possible causative agents, and secondly that a proportion of encephalitides, such as those due to VZV, measles virus or *Mycoplasma pneumoniae*, are post infectious and the causative agent might not be identifiable by the screening tests employed. In addition not all encephalitis cases are caused by microorganisms. The proportion of aetiologically confirmed acute encephalitis cases has varied in the past from 15–100% [23,25,28,30]. The commonest identified cause was HSV, with a relatively consistent incidence across the globe probably reflecting a lack of geographical specificity for herpes viruses.

Reliance upon passive systems of encephalitis reporting by healthcare providers and diagnostic laboratories may lead to under-reporting, even where aetiology has been confirmed [19,20]. Similarly, under-reporting may also occur either because acute encephalitis is not a notifiable disease, or as a result of variation in practice between countries [9]. Regional variation within countries in the proportion of hospitalisations where aetiology was confirmed also occurred, perhaps reflecting variation in clinical and laboratory diagnostic practice [20]. For some clinicians, confirmation of aetiology may not be seen as a priority, especially if all suspected cases of acute encephalitis are routinely treated with acyclovir [20].

Accurate aetiological diagnosis is required to increase the usefulness of surveillance of acute encephalitis, especially in view of concerns about new and re-emerging infections [7,19,42].

### Study type and reliability

Of the 25 studies reviewed, only 12 covered all ages. Of these, 1 was longitudinal observational [5], 2 were prospective [4,35]. Of these 2 were from Western settings and one from Thailand [35]. Of the 12 studies covering all ages, 5 were population-based [18,20,21,34,35], 5 were hospital surveillance, admission, or discharge-based [4,5,13,19,36] and 2 analysed the response from a uniform questionnaire sent to all relevant institutions within the study population [17,37]. Although prospective, observational or interventional study design is optimal, the majority of the studies reviewed could not be conducted in this way for logistical reasons. An advantage of using hospital discharge data is that most patients with encephalitis were likely to be hospitalised because of the severity of the illness [19]. However some patients may die before reaching the hospital, thus not being included. Population-based studies in the past have not provided good estimates of the overall disease burden of acute encephalitis because they either focused on specific pathogens or were conducted in small populations [36,43]. Retrospective studies [15,18] can only review cases that have been confirmed as acute encephalitis of any cause, within a specified time-limit and so analyses will always be dependent on the quality of record keeping during the study period, clinical case definitions applied and laboratory criteria employed at the time of diagnosis. Recall bias and small sample sizes may have also significantly hampered these retrospective studies [15,18] including the possibility of an over or under estimation of acute encephalitis incidence.

### Vaccination

The epidemiology of acute encephalitis has changed substantially over the years with the introduction of vaccines. In particular, a dramatic change in the aetiology of childhood encephalitis in Finland has been observed since vaccination programmes eradicated measles-, mumps- and rubella-associated encephalitides [44]. Similar effects have been reported in other studies based in the West [12,36] and in the tropics where JE vaccinations have been used [25].

### Incidence rates of acute encephalitis in Western Industrialised countries

Most of the studies of acute encephalitis of any cause have emerged from Western countries, where HSV, measles, mumps or rubella viruses were the most commonly identified causative agent. HSV has been known to be an important cause of encephalitis for some time [45]. Its rapid identification and effective treatment is crucial, particularly amongst middle-aged and elderly people who appear most vulnerable to its sequelae [46]. Of concern are reports of an increase in arthropod-borne viruses in Western countries [8,18]. WNV outbreaks in North America, Southern Europe, Africa and Asia [47] have drawn attention to the potential for spread of mosquito-borne viruses [42,48].

The 19 studies set in the West have a range of annual incidence from 1.4–13.8 per 100,000. Children only were studied in 9 reports [10-12,14-16,39-41,49], 4 studied adults only [7,9,33,38], and 12 studied all ages [4,5,13,17-21,34-37]. One study gave incidence figures for both adult and paediatric populations [20]. One study reported encephalitis mortality only [34]. In the prospective study from a Western setting looking at all age groups, which included arboviral infections, the incidence of encephalitis was 7.4 [4].

In the paediatric studies the incidence of encephalitis ranged from 2.37 to 14.25 per 100,000 overall, changing to 10.5 to 13.8 per 100,000 if only the 2 prospective studies are included [11,14], giving a minimum incidence of 10.5 per 100,000 [12]. Both of these studies were from Western countries.

In the adult studies, the lowest reported incidences were 0.9 [33],1 [38],1.4 [7], and 2.2 [9] per 100,000. All of these were single-centre hospital-based prospective studies with relatively small sample sizes. In the 12 studies including all age groups the incidence per 100,000 ranged from 1.5 to 9.25 [4,5,13,18-21,34-37]. The two studies from a Western setting of all age groups, with incidences of less than 2.0 per 100,000 were both retrospective analyses of national hospital episode statistics or medical databases from England and France [20,21]. Given that these are dependent on the discharge coding diagnoses, which are notoriously unreliable, it is perhaps not surprising that they gave a lower incidence than the prospective hospital-based studies.

The two studies in adults with incidences of 1.0 or less per 100,000 were both prospective studies from a tropical setting, one from Libya and one from Nigeria. Both studies included all CNS infections and then subdivided them by diagnosis. In the discussion for both studies the authors are careful to explain the lack of viral diagnostic facilities and cite this as a possible reason for the relatively low incidence of encephalitis in their studies [33,38]. Whilst this is a valid concern, their findings should not be dismissed. Even in the case of JE where incidences can reach 389 per 100,000 during outbreak situations [50] the reported incidence can be as low as 0.4 per 100,000, in JE endemic areas where vaccination has not occurred, if the diagnostic capabilities are limited [51].

### Incidence of Encephalitis in Tropical Countries

In recent years, the epidemiology and distribution of JE has changed [52]. The disease incidence is decreasing in China, Japan and Korea, and Thailand where it appears to be associated with widespread vaccination campaigns; in contrast it appears to be increasing in parts of Bangladesh, Burma, India, Nepal, and Vietnam [16,23,25,31,53,54]. Although the reasons for these changes are not clear, they may include the adoption of rice cultivation, establishment of larger pig farms and promotion of pig-breeding as a food source, and possibly climate changes [52]. Data from tropical countries successfully implementing vaccination, would suggest that even where JE incidence has fallen as a result, the minimum overall incidence of viral acute encephalitis in JE endemic areas is similar to that in Western industrialised countries [26,27,31], with the reported incidence of JE in the tropics ranging from 2 to 15 per 100,000.

## Conclusion

From the 25 studies reviewed in detail, in the most robust, prospective studies conducted in Western industrialised and tropical countries a minimum incidence of 10.5 per 100,000 AES cases is reported for children and 2.2 per 100,000 for adults and 6.34 per 100,000 for all ages. For ease of use in protocols and as benchmarks we therefore suggest that the 2 per 100,000 be used as the minimum incidence for adults, and 10 per 100,000 for children and 6 per 100,000 for all age groups for use in future WHO AES surveillance standards.

The fact that the aetiology of acute encephalitis remains unknown in the majority of patients [15] represents a diagnostic challenge for the future. The increasing incidence of encephalitis due to emerging agents, such as JEV, WNV [19], TBEV [15], and *Mycoplasma pneumoniae *[49], will present additional challenges. This review shows that in most studies conducted in both Western industrialised and tropical countries, an overall incidence of acute encephalitis of between 1 and 15 per 100,000 is reported, with higher incidences in children than adults. There were no prospective studies from tropical settings looking at the incidence of acute encephalitis in children only.

## Methods

A series of literature searches were performed using three search engines (PubMed, OVID and EMBASE) to identify epidemiological studies that estimate the incidence of AES in Western industrialised and tropical countries (Table 1). The search results for each database were assessed for duplicate articles. Studies were considered further if they (1) performed population-based surveillance for encephalitis cases, AES, or specific infectious aetiologies of encephalitis in a well-defined geographic area and time frame, or (2) performed sentinel or hospital-based surveillance for the above diagnoses or syndrome in a geographic area and time frame for which the population could be estimated and an incidence calculated. The abstracts of acceptable articles were read and those that matched our criteria were analysed further. Additionally, articles already known to the authors, or cited by articles identified during the above literature search, were also retrieved, assessed and included if appropriate.

In summary, 7,653 articles were retrieved from Pubmed, 1,419 were retrieved from OVID and 3,364 were retrieved from EMBASE using the above search criteria. This totalled 12,436 articles at the time the search was carried out in June to August 2008. In view of the large number of retrieved articles, the authors carried out an initial screening by title to select only the articles most applicable to our inclusion criteria and to remove duplicates. Abstracts of 1,083 articles with relevant titles were reviewed further together with other articles already known to the authors and matching the inclusion criteria or referenced by other articles from the database search. Of these, 87 articles were considered relevant. These, were read through fully. 25 articles exactly matching our criteria were thus included for study with an additional 62 included as they gave values for incidences of viral encephalitis due to specific viruses. Papers in which incidence data were not given but where there was sufficient data given in the paper for the values to be calculated were also included. All incidence figures are presented as incidence per 100,000 population for ease of comparison between them. Articles were excluded throughout for a number of reasons; some did not discuss encephalitis despite being retrieved through the 'encephalitis' keyword. Others discussed laboratory markers rather than incidence in a given population; including several articles claiming to discuss encephalitis epidemiology, which despite providing good details on their study design, lacked actual incidence figures.

## Competing interests

TS was on the WHO expert panel that produced the Japanese Encephalitis Surveillance Standards.

## Authors' contributions

MF and TS conceived of the review. PL, FJ and HCAE wrote the review, TS and MF provided substantial intellectual contributions to the review.

## Supplementary Material

Additional file 1 **This file gives further details of the selected papers containing encephalitis incidence data.** Click here for file
