# Supplementary material for: The incidence of acute encephalitis syndrome in Western industrialised and tropical countries
Source: Virol J. 2008 Oct 30;5:134. doi: 10.1186/1743-422X-5-134 (PMC2583971; doi:10.1186/1743-422X-5-134)
Supplement: Additional file 1 — This file gives further details of the selected papers containing encephalitis incidence data. [file 1743-422X-5-134-S1.doc]

| **Reference** | **Setting Western/ Tropical (W/T); Study year SY; No of Cases (N=); Study type; Population under study** | **Aetiology** | **Exclusion Criteria** | **Aetiologies included** | **Clinical definition of AE case** | **Laboratory Diagnostic criteria for AE case** | **Annual Incidence**  **(data converted to / 100 000 population*)**  **incidence calculated from data in paper**** | **Notes** |
| --- | --- | --- | --- | --- | --- | --- | --- | --- |
| Klemola et al (1965), Kaeaeriaeinen et al (1964) [1, 2] | **Helsinki, Finland (W)** (SY) 1945-1963, N=108, Prospective, All ages | All causes | not specified | all encephalitis | Same criteria as used by Meyer et al 1960 (Meyer, H.M., Jr., et al., Central nervous system syndromes of "viral" aetiology. A study of 713 cases. Am J Med, 1960. 29: p. 334-47). | After 1952 viral studies gradually introduced, 1952-58 virus isolations, 1957 CF against parotitis and poliovirus types 1-4, influenza A &B, adeno, herpes simplex, Eaton agent, parainfluena, reovirus, ornithosis, choriomeningits lymohcytica (LCM) Coxsackie B3 and 5, encephalomyocarditis and respiratory syncitial virus. Since 1957 HI for TBE, Neutralization tests for ECHO 5, 6 and 9, | 2 to 3 |  |
| Beghi EA , et al. (1984) [3] | **Minnesota US (W)**  SY:1950 – 1981 N=189 Prospective Population Based All ages | All causes | If diagnosed with other diseases eg non-inflammatory conditions or lab findings suggestive of non-viral infection. | All causes but California virus, Mumps and HSV were most commonly detected | “Confirmed”: Clinically, in absence of another diagnosis or in presence of another inflammatory disease of Acute/subacute onset + pleocytosis >5/mm^3^, fever, meningeal signs. Pathologically, as result of post mortem. “Possible”: ill defined neuro signs or inability to exclude other diseases. | Considered viral cause if: 1) Virus cultured from CSF; 2) Complement fixation (CF) or haemagglutination inhibition (HI) showed fourfold change in serum/CSF antibody titre in acute and convalescent phases; 3) Counter-immuno-electrophoresis was positive | 7.4 (age and sex adjusted rate) 8.1 (both sexes)  6.7 (females)  9.7 ( males) | Various vaccines given to some patients prior to onset of encephalitis |
| Henrich, et al. (2003) [4] | **Thailand (T)** SY 1993-1998, N=3777 all ages prospective | All causes |  | All clinical presentations of AES | Patient history, physical examination and lab data Presence of confusion, alteration of consciousness, fever, nausea, headache, muscle spasms or seizures | Blood count CSF profile, cell count and differential, latex agglutination tests, bacterial culture and sensitivity assay on CSF viral diagnostics including capture ELISA for JE and dengue | 6.34 |  |
| Ponka, et al (1982) [5] | **Helsiniki, Finland** SY 1980 N=9 Retrospective All Ages | All causes | not specified | All CNS infections numbers given for meningo-encephalitis and encephalitis | not specified | CSF Pleocytosis >10per mm3 | 3.5 |  |
| Nicolosi (1986) [6] | **Olmsted County USA (W)** SY 1950-1981 N= 52, Retrospective, All ages | All causes | poliomyelitis and non viral causes | all cases of CNS infections Viral encephalitis as sub category | Viral infection with evidence of brain parenchymal involvement. Clinical confirmation included acute or subacute onset inflammatory disease with a combination of fever , meningeal signs and CSF pleocytosis of > 5 WBC/ mm3 | Virus isolation from the lesion or a viral culture of CSF or CF or HI with a 4-fold increase or decrease in titre of antibody between acute and convalescent sera or positive CIE test to detect California virus infection. HSV, Mumps, Coxsackie B virus, Echovirus, Enterovirus, Influenza virus, Rubella, Measles, Varicella | 7.4 (adjusted incidence rate),    22 (children <1yr),  15 (1-4yrs),  30 (5-9yrs),  6(10-19yrs) |  |
| **Reference** | **Setting Western/ Tropical (W/T); Study year SY; No of Cases (N=); Study type; Population under study** | **Aetiology** | **Exclusion Criteria** | **Aetiologies included** | **Clinical definition of AE case** | **Laboratory Diagnostic criteria for AE case** | **Annual Incidence**  ***(data converted to / 100 000 population)**  **incidence calculated from data in paper**** | **Notes** |
| Pedersen (1956)[7] | **Jutland (W)** SY 1952-1954 N= 135 (1952), N=183 (1953), N= 185 (1954) Retrospective, All ages | All causes | smaller centres not contacted | questionnaire survey to main centres in Jutland | not specified | Widal, Weil & Paul Bunnell tests, some samples tested for Q fever toxoplasmosis virus culture attempted unsuccessfully in a minority of cases. CSF cell count, glucose albumin and protein | 6.75 **(1952)  9.15 **(1953)  9.25 **(1954) | in context of an epidemic of unknown aetiology Jutland popn 2 million) |
| Khetsuriani, et al. (2002) [8] | **USA (W)**  SY: 1988- 1997 N=186,804 Retrospective, All Ages | All causes | Not accurately specified, but implied by strict adherence to WHO ICD codes | All, but HSV, VZV, arboviruses & toxoplasma were most common | Based on WHO 9^th^ edition ICD codes where at least one of the ICD codes was listed on discharge notes National Hospital Discharge Survey Data | Not specified | 7.3 |  |
| Khetsuriani, et al. (2007) [9] | **USA (W)**  SY 1988-1997 N=186 804 SE +/- 10 482) Retrospective all ages | All causes |  | All caused of encephalitis specifically looked at HIV associated deaths | ICD-9CM encephalitis code | not specified | 7.3 (all ages hospitalizations)  13.7 (<1yr ),  5.1 (1-4yrs ),  4.1 (5-19yrs,  8.1 (20-44yrs ),  7.1 (45-64yrs ),  10.6 (>=65 ) |  |
| Kamei et al, (2000) [10] | **Japan (T)** SY 1989-1991 Cases, all Ages nationwide questionnaire survey | All causes |  | all | Encephalitis included meninogo-encephalitis and meningo-encephalomyelitis. | not specified | 17.7+/- 3.3 (all encephalitis)  5.5+/-11.0 (viral encephalitis)  3.5+/-1.0 (HSV encephalitis) |  |
| Trevejo, (2004) [11] | **California, US (W)**  SY: 1990- 1999 N= 13,807 Retrospective All ages | All causes | Patients with “AIDS” | All, but majority of cases were of unspecified aetiology | Not specified in detail but based on WHO ICD 9^th^ edition hospital discharge data | Not specified in detail but based on WHO ICD 9^th^ edition | 4.3  4.2 (males)  4.5 (females) | Exclusion of male AIDs patients: ?likely difference in male: female incidence |
| Laursen, et al (2003) [12] | **Denmark** SY 1994- 2000 N=29 all ages retrospective study | All causes |  | non-bacterial meningitis, non-bacterial encephalitis, encephalomyelitis or myositis | symptoms of meningitis, with fever headache, nausea vomiting and vertigo , on meningo-encephalomyeltiis no further details | IgM and IgG against TBEV in serum and or CSF | 3.81 (meningitis and encephalitis and meningo encephalitis combined in incidence) |  |
| **Reference** | **Setting Western/ Tropical (W/T); Study year SY; No of Cases (N=); Study type; Population under study** | **Aetiology** | **Exclusion Criteria** | **Aetiologies included** | **Clinical definition of AE case** | **Laboratory Diagnostic criteria for AE case** | **Annual Incidence (data converted to / 100 000 population*) incidence calculated from data in paper**** | **Notes** |
| Mailles, et al. (2007) [13] | **France (W)**  SY: 2000-2002 N= 1,200 Retrospective All ages | All causes | Patients infected with HIV | All, but HSV & VZV most common | Not specified in detail. Study used WHO ICD codes (10th Edition) | Not specified in detail but study does mention WHO ICD codes (10th edition) | 1.9 |  |
| Kupila, et al. (2006) [14] | **Finland (W)**  SY: 1999-2003 N= 42 Prospective Adults ≥16years | All causes | Evidence of vascular, malignant, metabolic, psychiatric, demyelinating, toxic or traumatic aetiology | All, but VZV, HSV-1 and TBE were most common | ≤3weeks duration of symptoms & signs of cerebral involvement, altered consciousness/ personality, epileptic seizures or focal neurological signs +CSF WBC count>5×10^6^ /L or neuroradiology/EEG findings compatible with AE. | “Confirmed”: microbe / microbe nucleic acid detected in CSF or specific IgG/IgM antibodies in CSF. “Probable”: serum seroconversion or if serum IgM antibodies for one specific microbe were observed | 2.2 |  |
| Radhakrishnan et al (1987) [15] | **Libya (T)** SY 1983-1984, N=5 Prospective Adults > 15yrs old | All causes | Aseptic meningitis, acute bacterial meningitis, TB meningitis. Neurosyphilis, Neurohydatidosis, bilharzial myelopathy | All encephalitis | Clinical evidence of more deep-seated neurological involvement such as coma, convulsions, personality changes and pathological reflexes. | CSF cell count and microscopy, protein and glucose, Bacterial culture, VDRL, TPHA in selected cases EEG and CT viral confirmation not available | 1 |  |
| Nwosu et al (2001) [16] | **Nigeria (T),** SY October 1991-October 1993 N=2 Prospective Adults >=16 years | All causes | rabies encephalitis, acute bacterial meningitis, aseptic meningitis, chronic non-TB meningo-encephalitis | all non-rabies encephalitis | AES criteria as described by Adams and Victor 1986 Principles of Neurology3rd Edition | CSF analysis for protein glucose cellular content, cultures for bacterial isolation and Ziehl Nielsen stains for acid-fast bacilli. Gram stains and India ink stains in spun CSF. Blood cultures. Blood and CSF serological examinations venereal diseases research laboratory (VDRL), Treponema pallidum haemagglutination test (TPHA), HIV 1&2 immunocombi spot test. Other viral studies and isolation not done. Routine biochemistry. EEG in selected patients | 0.9 (non-rabies encephalitis) |  |
| **Reference** | **Setting Western/ Tropical (W/T); Study year SY; No of Cases (N=); Study type; Population under study** | **Aetiology** | **Exclusion Criteria** | **Aetiologies included** | **Clinical definition of AE case** | **Laboratory Diagnostic criteria for AE case** | **Annual Incidence**  ***(data converted to / 100 000 population)**  **incidence calculated from data in paper**** | **Notes** |
| Rantalaiho, et al. (2001) [17] | **Finland (W)**  SY: 1967-1991 N=322 Prospective one hospital in Helsinki Adults ≥ 15 yrs | All causes | Evidence of vascular, malignant, metabolic, psychiatric, demyelinating, toxic or traumatic aetiology. Also excluded if admitted to other than Helsinki University Central Hospital | All, but HSV, VZV, Influenza A and Mumps were most common | Symptoms of acute CNS involvement of ≤4wks duration eg disturbances of brain function, lowered level of consciousness or focal neurological signs. Combined with abnormal findings of CSF ± EEG | Sera obtained in acute phase & 2-3wks later for measuring CF to most viruses. For TBE & rubella suspected cases, virus-specific serum IgM determined. “Confirmed”: virus detected in CSF/brain biopsy while other antibodies remained negative. “Probable”: seroconversion/four-fold increase in serum antibody levels/relevant clinical disease within 4wks of CNS symptoms | 1.4 | National Mumps vaccination programme implemented in 1982 |
| Davison, et al. (2003) [18] | **England (W) PY 2003** SY 1989-1998 N=6414 all ages children N=2734 Retrospective All ages | All causes |  | HSV (most common), VZV, CMV, EBV, MMR, | Definition not accurately specified, but clinical diagnosis was based on fever, headache and altered mental state and WHO ICD codes | Based upon clinical evidence using the WHO ICD 9^th^ and 10^th^ edition codes and laboratory data (where any virus under study was detected in CSF) | 1.5 (2.8 in children)  (1.1 in adults) |  |
| Rantakallio et al (1986) [19] | **Finland (W)** SY 1966 to 1972 N= 21 viral encephalitis from 12 000 birth cohort 1966 children < 14 years | All causes | only children in the birth cohort of 12000 followed from 1966 | all | Combination of symptoms, signs and lab results, including EEG, elevated CSF protein and leucocytes, duration of illness and convulsions | not specified | 12.6 (viral encephalitis),  688.0 (viral CNS infections |  |
| Wang et al (1981) [20] | **Halifax Canada (W)** SY January 1972-April 1980 N=180 Viral CNS infections N= 76 viral encephalitis Retrospective Ages, <=16 years | All causes | Prior antibiotic therapy, incomplete medical records or other explanatory conditions N=69 | all viral CNS infections | Not detailed but included convulsions and coma | CSF Virus isolation and PCR, Serology for: Mumps, Varicella, HSV, enteroviruses (coxsackie virus, echovirus, picornovirus) measles etc. CT and EEG in selected cases | 19.5 ( all viral CNS infections) 8.2** (viral encephalitis ) |  |
| **Reference** | **Setting Western/ Tropical (W/T); Study year SY; No of Cases (N=); Study type; Population under study** | **Aetiology** | **Exclusion Criteria** | **Aetiologies included** | **Clinical definition of AE case** | **Laboratory Diagnostic criteria for AE case** | **Annual Incidence**  ***(data converted to / 100 000 population)**  **incidence calculated from data in paper**** | **Notes** |
| Wong et al (1987) [21] | **Hong Kong (T)** SY 1975-1986 N= 57, Retrospective, Ages <14 years | All causes | Viral meningitis without signs of encephalitis bacterial, fungal and tuberculous meningitis, cerebral abscess, cerebral haemorrhage or infarction, Reye's syndrome, metabolic or toxic encephalopathy. | all | Clinical features of inflammation of the brain and or meninges such as fever, convulsion, change in sensorium, neck rigidity of positive Kernig's sign | Examination of CSF, EEG, Radio-nuclide brain scan and computed tomography (CT)of brain in some cases. Viral serology using CF antibody or IF methods for antibodies against common viruses such as influenza A and B, Coxsackie virus, adenovirus, JE, HSV, measles, mumps rubella, enterovirus lymphochoriomeningitic virus and CMV. CSF tested in acute phase for similar viral antibodies and cultures. Oropharyngeal secretions and rectal swab and stools for viral culture. | 14.25** |  |
| Ilias, et al. (2006) [22] | Crete, **Greece (W)**  SY: 2000-2004 N= 18 Prospective Children <14yrs | All causes | Children with a post-varicella presentation of cerebellitis | All, but Echovirus, HSV-1, VZV, CMV & influenza A most common | Not specified. Mentions common presentations: altered consciousness, coma, motor deficits, cerebellar dysfunction, convulsions and speech disorders. CSF and neuroimaging also used | Not specified in detail but based on blood and CSF serology rather that detection of causative agent by PCR/culture. Refers to other common studies’ lab criteria.(Cizman et al, Davison et al, Khetsuriani et al, Kolski et al) | 2.6 |  |
| Ishikawa, et al. (1993) [23] | Ishikawa (1993) **Japan (T)**  SY: 1984-1990 N=256 Retrospective Children <15years only | All causes | Infections involving the spinal cord | All, but measles and herpes were most common | Based on individual paediatrician’s judgement questionnaire | Not accurately specified | 3.3  3.9 (boys)  2.6 (girls) | Results from 1984 were excluded due to possible under-reporting |
| Rantala, H. and M. Uhari (1989) [24] | **Finland (W)** (1989) SY 1973-1987 N= Retrospective <16 yrs only | All causes | BM, Reye’s syndrome/ other metabolic encephalopathies, taking immuno-suppressants | All, but VZV, Mumps, HSV and Measles most common | ≥1 of following in conjunction with current infectious disease: Reduced conscious level, Mental deterioration, Motor / sensory disturbances | Complement fixation & Haemagglutination inhibition used. Four-fold or more increase in serum antibodies / titre >1:64 was diagnostic, if virus/virus antigen isolated from CSF, or nasopharynx | 8.8 | MMR vaccine started in 1982, no AE cases due to MMR after 1982 were seen |
| **Reference** | **Setting Western/ Tropical (W/T); Study year SY; No of Cases (N=); Study type; Population under study** | **Aetiology** | **Exclusion Criteria** | **Aetiologies included** | **Clinical definition of AE case** | **Laboratory Diagnostic criteria for AE case** | **Annual Incidence**  ***(data converted to / 100 000 population)**  **incidence calculated from data in paper**** | **Notes** |
| Kolski, et al. (1998) [25] | **Toronto, Canada (W)**  SY: 1994 – 1995 N=145 Prospective Children 1 month -18 years | All causes | Bacterial infections, viral exanthema, non-infectious disorder. Also <1 month old, ≥18years old, previous immuno-suppressive / neurological disorder | All, but  *M. pneumoniae* and HSV were most common | Depressed/altered level of consciousness ≥24hrs, including lethargy, extreme irritability or significant change in behaviour or personality. Encephalitis defined as encephalopathy + ≥2 of: 1)Fever ≥38°C, 2)Seizures, 3)Pleocytosis, 4)Abnormal Imaging | “Probable”: serological detection of IgM antibodies plus >four-fold change of titres in paired serum samples or single high titre of CF antibody to *M.Pneumoniae* of ≥1:64. “Confirmed”: pathogen detected in CSF or brain tissue sample (culture or PCR analysis) AND serological evidence of acute infection (as per “Probable”) | no data |  |
| Cizman, et al (1993) [26] | **Slovenia (W)**  SY: 1979- 1991 N=170 Retrospective Population based Children: 1 month-15 years | All causes | Symptoms of meningitis or abnormal CSF | All but VZV, HSV, Measles, Mumps, Rubella, *Chlamydia psittaci* & TBE most common | Not specified, but indicated “children whose clinical manifestations were compatible with a picture of AE syndrome, where other local/generalised neurologic involvement was ruled out”. | Based on virus isolation ± serological tests. TBE: serum antibody titres ≥1:128. HSV: antibody titre at serum:CSF ratio ≤20:1 | 6.7 |  |
| Koskiniemi, et al. (1989) [27] | **Finland (W) PY: 1991**  SY: 1968- 1987 N=462 Prospective, admissions to 1 hospital in Helsinki Children: 1 month - 16 yrs only | All causes | Patients with other diseases especially purulent, systemic, vascular and neoplastic. Neonatal infections (0-4wks) | All but most common were VZV, *M. pneumoniae*, respiratory and adenovirus | Neuro symptoms lasting <4wks eg lowered consciousness, foal/generalised seizures, opisthotonos, pareses, tremors, ataxia, hypotonia, mental changes, impaired speech, dizziness, diplopia | “Confirmed”: virus/antigen detected from brain tissue/CSF, or serum:CSF antibody ratio ≤20. “Suggested”: ≥four-fold rise in antibody titre in paired serum specimens or high titre (≥64) in 1 serum specimen, or positive viral culture/ antigen finding in extracranial source. | 8.3 (average)  (range 19.8 in 1974 to  2.5 in 1985 and 1986) . | 1983 MMR vaccine introduced. Also Ann Neurol 1991 |
| Koskiniemi, M., M. Korppi, et al. (1997) [28] | **Finland (W)**  SY: 1993 – 1994 N=175 Prospective Children 1month – 15yrs | All causes | Patients with other diseases especially purulent, systemic, vascular and neoplastic diseases. Neonatal infections (0-4wks) | All, but VZV, respiratory and adenovirus most common. Rotavirus, EBV and HSV also important | Acute onset (<4wks) of symptoms of focal or generalised brain involvement eg pareses, sensory symptoms, convulsions, linguistic or mental dysfunction. Diagnostic if lasting >24hours. If <24hrs, EEG and CSF findings taken into consideration | “Confirmed”: organism / nucleic acid / specific IgM antibodies detected in CSF or if ratio of IgG antibodies in serum:CSF was ≤20. “Suggested case”: if seroconversion occurred/rise in antibody titre in paired specimens, specific IgM/high IgG titre ≥110EIU in 1 specimen, specific IgGs to organism in CSF, positive viral culture/antigen in extracranial source or if chickenpox/vaccination in 4 wks before symptoms | 10.5 | change in aetiology of encephalitis since mass vaccination programs for mumps was introduced |
| **Reference** | **Setting Western/ Tropical (W/T); Study year SY; No of Cases (N=); Study type; Population under study** | **Aetiology** | **Exclusion Criteria** | **Aetiologies included** | **Clinical definition of AE case** | **Laboratory Diagnostic criteria for AE case** | **Annual Incidence**  ***(data converted to / 100 000 population)**  **incidence calculated from data in paper**** | **Notes** |
| Bond, et al. (1965). Quick, et al. (1965) [29, 30] | **Florida USA (W)** SY 1962 Prospective outbreak investigation All ages | All causes (SLE) | Not specified | all cases of encephalitis | headache, personality changes, confusion and disorientation, stupor or coma, tremors of face and hands and nuchal rigidity | HAI EEE, WEE,SLE, MVE dengue II, then limited to HI and CF for SLE | 30.8 |  |
| Leake, J. P. (1933) [31] | **St Louis, USA PY 1933** SY 1933 Retrospective all ages | All causes (SLE) | Not specified | all cases of encephalitis but implied from paper majority assumed to be SLE | not specified | not specified | 99 | presumed first reports of what is now called SLE |
| Chakrabarty et al (1986) [32] | **Uttar Pradesh, India (T)** SY 1985 N=309 Gorakhpur District N=532 Deiora District, Prospective outbreak investigation, All ages | All causes but majority JE | Purulent meningitis and falciparum malaria | All encephalitis but presumed to be JE implied from paper | High fever, headache, stupor, disorientation, meningeal signs, tremors, coma. Presumptive diagnosis of JE | 5/ 8 single sera samples tested had a titre between 80-160 for JE and or WN none of sera reacted to Group A antigens | 80( Gorakhpur),  160/( Deiora District) |  |
| Yamada, et al (1971) [33] | **Thailand (T)**  SY 1969-1970, Prospective outbreak investigation, All ages | All causes but majority JE |  | all encephalitis but majority in 1969 and 1970 JE | Clinical manifestations seen: Disturbance of consciousness, difficulty in swallowing and convulsions. | Neutralisation tests, Haemagglutination inhibition tests, virus isolation, Serology, Fluorescent antibody technique, electron microscopy, used to confirm presence of JE , majority of cases diagnosed clinically only. | 2-5 (1965-1968 pre JE epidemic)  20.3 (1969 JE)  14.4 (1970 JE) | in context of JE epidemic |
| Vajpayee, et al (1992) [34] | **India (T)** SY July 1989 August 1989 N= 140 Prospective outbreak investigation, All ages | All causes but majority JE | Not specified | all cases of encephalitis but implied from paper majority assumed to be JE | Clinical features described include, fever and headache, acute stage neurological manifestations like neck rigidity, positive Kernig's sign, exaggerated reflexes, extensor plantar responses, convulsions, disorientation, semi consciousness, unconsciousness and abnormal movements | Serum samples from7 acute and 8 convalescent cases remaining cases presumptive diagnosis f JE made | 389 ** |  |
| **Reference** | **Setting Western/ Tropical (W/T); Study year SY; No of Cases (N=); Study type; Population under study** | **Aetiology** | **Exclusion Criteria** | **Aetiologies included** | **Clinical definition of AE case** | **Laboratory Diagnostic criteria for AE case** | **Annual Incidence**  ***(data converted to / 100 000 population)**  **incidence calculated from data in paper**** | **Notes** |
| Narasimham et al (1988) [35] | **Uttar Pradesh, India (T) PY 1988** SY 1988 N= 4544 Retrospective, All ages | All causes but majority JE | non JE | Encephalitis implied from paper presumed JE | not specified | not specified | 18 |  |
| Kar et al (1998) [36] | **India (T) PY 1998** SY 1994, N=26 Karnal N= 14 Kurukshetra, Retrospective, All ages | All causes but majority JE | Not specified | All encephalitis but presumed to be JE | Fever, altered mental state/ coma and in addition some cases had convulsions, vomiting, cough and chest pain | not specified | 3.8 (Karnal ),  1.6 (Kurukshetra) |  |
| Rubin et al (1970) [37] | **Ilinois, USA (W) PY 1970,** SY 1969, N=25, Prospective outbreak investigation, All ages | All causes but majority SLE |  | All encephalitis presentations but majority SLE in context of epidemic | febrile illness with dysfunction of the CNS not obviously due to other pathologic processes with or without signs of meningeal irritation | HI test on all specimens, CF on most specimens for SLE | 196 | in context of SLE epidemic |
| Najioullah et al (2000) [38] | Najioullah(2000) **France (W) PY 2000** SY 1997 N=7 Prospective Lab based All ages | HSV | non HSV confirmed cases | HSV confirmed encephalitis only | Suspected CNS viral CNS infection and alteration of consciousness, (from mild lethargy to confusion, stupor or coma) focal neurological signs, (including bizarre behaviour, hallucinations and aphasia) and seizures | CSF PCR for HSV DNA positives confirmed by hybridisation and typed by second PCR | 0.233 |  |
| Skoldenberg et al (1984) [39] | **Sweden(W),**  SY March 1981 - Dec 1983, N=27 suspected HSVE, 53 confirmed Prospective Ages all> 4weeks | HSV | HSVE | confirmed HSVE and suspected HSVE | Signs and symptoms suggestive of acute encephalopathy, with fever, altered consciousness, changed personality, confusion, disorientation, seizures, dysphasia, hemiparesis, and or other focal neurological symptoms.  Randomised double blinded placebo controlled treatment trial | Brain biopsy if indicated, CT, EEG CSF and serum samples for HSV and measles IgM and IgG (IgA also in some cases)antibody by ELISA. CSF/ serum albumin rations VZV IgM and IgG antibodies in whom HSV not confirmed | 0.23** (Confirmed HSV encephalitis), 0.55** ( all suspected HSV encephalitis, ) |  |
| **Reference** | **Setting Western/ Tropical (W/T); Study year SY; No of Cases (N=); Study type; Population under study** | **Aetiology** | **Exclusion Criteria** | **Aetiologies included** | **Clinical definition of AE case** | **Laboratory Diagnostic criteria for AE case** | **Annual Incidence**  ***(data converted to / 100 000 population)**  **incidence calculated from data in paper**** | **Notes** |
| Hjalmarsson, (2007) [40] | **Sweden(W), PY 2007,** SY 1990-2001, N=236, Retrospective, All ages | HSV-1 & 2 | Obstetric admissions, admissions immediately preceding the HSE admission involving seizures or confusion. Admissions > 5years before the HSE diagnosis | HSV-1 also untyped HSV infection and HSV-2 infection | All hospital admissions with a primary diagnosis of HSE admissions with International Classification of Diseases 9th 054D, ICD 1oth B00.4, | Positive finding of HSV-1 by DNA PCR from CSF samples or detection of intrathecal HSV-1 antibody production. | 0.22 (HSV-1 only) 0.25 (HSV-1 and HSV-2 and untyped HSV infection) |  |
| Omland, et al. (2008) [41] | **Denmark (W) PY 2008** SY July 1999-Dec 2003 N=49 ,Retrospective, Ages > 15yrs | HSV-2 | Not specified | HSV-2 only | presence of 1 or more of the following : impaired consciousness, seizures or supranuclear neurological deficits. | HSV-2 PCR CSF anti HSV IgG relative to total IgG in CSF compared to serum | 0.03** (HSV-2 encephalitis ) 0.26 (all HSV-2 CNS presentations) |  |
| Grossman, et al. (1973) [42] | **Chiangmai, Thailand (T) PY: 1973** SY: 1970 N= 100 Prospective Population Based All Ages | JE | Alternate Diagnoses eg Reye’s, Meningitis, Diphtheria. Non-JEV aetiology | JE | Not specified accurately. Only “patients given clinical diagnosis of encephalitis by the attending physicians” | Diagnostic if HI titre rise of >four-fold between acute & convalescent specimens and >four-fold change in paired specimens for CF. No attempt made to isolate JEV from CSF/serum. Post-mortem used for virus isolation. | 14.7 (overall age and sex adjusted) 10.4 (for females)  19.1 (for males) | Outbreak of Dengue HF in 1969. No cases were reported in 1970 |
| Okuno, T., P. T. Tseng, et al. (1975) [43, 44] | **Taiwan (T) PY: 1975** SY: 1968-1971 N= 983 Prospective  All ages | JE | <1:10 serum antibody titre during course of illness as applied to results of HI tests | JE | Not stated. Physicians were asked to report suspected JE. | HI tests on sera were carried out in 2 phases:1st <5d after febrile onset, 2nd between 14th day and 6wks. “Confirmed”: paired sera ≥4-fold upward conversion with peak titre ≥1:320 or single serum ≥1:640 | 5.00 (in 1965) 7.70 (peak incidence reached in 1967) 2.48 (in 1971: 4th year after vaccine) | JE vaccine introduced in 1968. |
| **Reference** | **Setting Western/ Tropical (W/T); Study year SY; No of Cases (N=); Study type; Population under study** | **Aetiology** | **Exclusion Criteria** | **Aetiologies included** | **Clinical definition of AE case** | **Laboratory Diagnostic criteria for AE case** | **Annual Incidence**  ***(data converted to / 100 000 population)**  **incidence calculated from data in paper**** | **Notes** |
| Wu et al. (1999) [45] | **Taiwan (T) PY: 1999** SY: 1967- 1997 N= 277 Prospective Population based All ages | JE | Study only uses data to calculate incidence from Confirmed cases, not Suspected or Reported. Also excluded are non-JE causes | JE | “Suspected case”: fever ≥38°C and 1 of following:*Meningeal signs ± N+V and headache *Cortical irritation eg twitch, convulsion *Disturbance of consciousness *Cranial nerve symptoms *Pyramidal & EP signs. “Reported”: suspected case reported by physician. “Confirmed”: reported cases confirmed by serology | Paired serum samples taken in acute and convalescent phases with HI used for serology testing. Confirmed if Haemagglutination inhibition test for JE Serum sample collection lowest in 1966 and 1967 (49% and 55%) 80% coverage of cases in remaining years  1)HI titre of convalescent serum ≥1:160 with ≥four-fold rise in acute phase serum OR  2)HI titre of either single serum is ≥1:320 | 80% from 1975 not JE therefore estimate AES at 1.5/100 000 (including JE cases)  2.05 (in 1967)  0.03 (1997) | Pre 1967, reporting of cases was passive and retrospective. At start of study, surveillance systems were put in place 1968, mass vaccination against JE |
| Chunsuttiwat, S. (1989) [46] | **Thailand (T) PY: 1989**  SY: 1983-1989 N= 1500-2500 Prospective Routine disease surveillance All ages | JE | Not specified | JE | Not specified | 15-20% of all clinically diagnosed cases were serologically tested. Method of testing not mentioned | 2 to 5 (due to variation in Thai Provinces) | Vaccination against JE introduced in mid 1980s |
| Wang, et al. (2007) [47] | **Yuncheng, Shanxi Province, People's Republic of China (T) PY 2007** SY. July - August 2006 n= 66 Prospective outbreak investigation All ages | JE | Not specified | JE only | not specified | IgM to WNV IgM-capture ELISA kit (PanBio, Brisbane, Queensland, Australia) IgM to dengue virus JEV by JE-Dengue IgM Combination ELISA kit (PanBio). Results for JEV confirmed by JE Virus IgM-Capture ELISA kit (Shanghai B & C Enterprise Development Co. Ltd, Shanghai, People's Republic of China). | 1.32 |  |
| **Reference** | **Setting Western/ Tropical (W/T); Study year SY; No of Cases (N=); Study type; Population under study** | **Aetiology** | **Exclusion Criteria** | **Aetiologies included** | **Clinical definition of AE case** | **Laboratory Diagnostic criteria for AE case** | **Annual Incidence**  ***(data converted to / 100 000 population)**  **incidence calculated from data in paper**** | **Notes** |
| Hsieh et al (1961) [48] | **Taiwan (T) PY 1961,** SY 1960 Retrospective N= 287 All ages | JE | Not specified | JE (All causes assumed majority JE) | Cases reported to Provincial Health Administration and cases reported by physicians (Names screened to prevent duplication) no clinical criteria specified | HI and CF tests for JE used no criteria specified |  |  |
| Partridge, et al (2007) [49] | **Nepal (T) PY 2007 SY** 2006 Kathmandu Valley and Terai Nepal, N= 1481 AES, N=292 JE lab confirmed Retrospective, All ages | JE | non-lab confirmed AES | lab confirmed JE | Acute onset of fever and a change in mental status (symptoms such as confusion, disorientation, coma or inability to talk) and or a new onset of seizures (excluding simple febrile seizures) or was clinically diagnosed as AES, JE or viral encephalitis or lab confirmed JE | JE antibody by IgM capture ELISA on a serum of CSF specimen. | 1.6 (Lab confirmed JE incidence in Terai and inner Terai )  2.1 (Kathmandu valley) , 3.1(Kathmandu valley <15yrs) and 1.6 (Kathmandu valley >15yrs) |  |
| Akiba, et al. (2001) [50] | **Nepal (T) PY 2001** SY 15 August to 10th Sept 1997 N= 1819 Retrospective All ages | JE | Malaria, bacterial meningitis & typhoid fever | JE | High fever, altered sensory, neck rigidity, and unconsciousness  Patients from 3 districts | JE specific IgM ELISA | 145 | in context of JE epidemic total population 1 228, 223 ie 27.9% of total population affected by JE in 4 months |
| Thongcharoen (1985) [51] | **Thailand (T) PY 1985** SY 1970-1984 N=24166, Retrospective, All ages (70% under 15 years) | JE | Not specified | JE | Clinical symptoms and signs included: Fever and headache, alteration of consciousness nuchal rigidity, Kernig's sign, hemiplegia and papilloedema | HI initially and from 1980 onwards JE MAC ELISA for IgM and IgG 80% of cases confirmed serologically | 2.9-5.0 |  |
| Hashimoto, et al. (2007) [52] | **Japan (T) ; PY 2007;** SY 2000-2005; Whole population using 2000 census data Retrospective | JE | only notifiable vector-borne diseases notified by public health centres to local government (prefecture) | JE listed separately other aetiologies listed were Dengue. Japanese spotted fever Lyme disease, malaria and scrub typhus. | National Epidemiological Surveillance of Infectious Diseaes (NESID) Taniguchi, K., et al., Overview of infectious disease surveillance system in Japan, 1999-2005. J Epidemiol, 2007. 17 Suppl: p. S3-13 | not specified | 0.04 (JE) |  |
| **Reference** | **Setting Western/ Tropical (W/T); Study year SY; No of Cases (N=); Study type; Population under study** | **Aetiology** | **Exclusion Criteria** | **Aetiologies included** | **Clinical definition of AE case** | **Laboratory Diagnostic criteria for AE case** | **Annual Incidence**  ***(data converted to / 100 000 population)**  **incidence calculated from data in paper**** | **Notes** |
| Paul et al (1993) [53] | **Saipan (T) PY 1883, SY** Sept 1990-28 Feb 1991 N=10 Retrospective All ages | JE | non JE | JE | Febrile illness, (temp>= 38oC), with one or more of: Headache, confusion, seizure or coma. | Fourfold rise in serum antibody titre to JE without a similar rise in antibody to dengue viruses by serum dilution neutralization or CF. | 25 |  |
| Kari et al. (2006) [54] | **Bali, Indonesia (T) PY: 2006**  SY: 2001- 2003 N=239 Prospective Population based Children <12yrs only | JE | Confirmed Bacterial Meningitis, cerebral malaria, tumours | JE | Acute onset of fever ± changes in mental status ± meningeal irritation ± any neurological deficits | “Confirmed case”: Detection of JEV-specific IgM antibody in CSF. “Suspected case”: detection of JEV-specific IgM in serum. | 7.1 |  |
| Hoke, et al. (1988) [55] | **Thailand (T) PY: 1988**  SY: 1984-1985 N= 11 Placebo-controlled, blinded, randomized trial Children aged 1-14ys only | JE | Not specified | JE | Fever >38°C, lethargy, obtundation or coma. Every admission with febrile CNS disease or haemorrhagic fever was examined. (JE vaccine induced encephalitis: occurrence of disease during 2 wks after immunisation with JE vaccine) | >5 leucocytes/mm^3^ in CSF. > 100 units of anti-JEV IgM antibody in CSF or serum and serum level of anti-JEV IgM > anti-dengue IgM antibody | 51 (in placebo group) 5 (in each vaccine group) | Study aimed to test efficacy of bivalent and monovalent JE vaccines |
| Vijayarani, et al (2000) [56] | **India (T)** SY 1991-93, N=1 all ages population of Thnjavur District Tamil Nadu, Prospective, Ages 5-12 yrs | JE | non-JE | all cases of Japanese encephalitis | not specified | Seroconversion measured by HI using JE, WNV and Dengue antigens HI results were confirmed using virus specific IgM antibody using MAC ELISA | 0.56** | JE present in area but outside of epidemic years. |
| **Reference** | **Setting Western/ Tropical (W/T); Study year SY; No of Cases (N=); Study type; Population under study** | **Aetiology** | **Exclusion Criteria** | **Aetiologies included** | **Clinical definition of AE case** | **Laboratory Diagnostic criteria for AE case** | **Annual Incidence**  ***(data converted to / 100 000 population)**  **incidence calculated from data in paper**** | **Notes** |
| Gajanana et al (1995) [57] | **India (T)** SY 1989-1990 N=229 Prospective survey children aged 5-9yrs | JE |  | encephalitis all JE predominantly implied from paper | not specified | HI against JE, WN and DEN-2, ELISA for WN, JE and DEN-2 | 150 (children aged 5-9yrs) |  |
| Tigertt, et al. (1957) [58] | **Japan (T)** SY: 1946-1949 N= 40 Longitudinal population based Young children. By study end, children were aged 5-10 yrs | JE | Not specified | JE | Not specified | Not specified | 41.2 ,  11.8 (in vaccinated)  57.0 (non-vaccinated) |  |
| Grayston et al (1962) [59] | **Taiwan (T)** SY July 1955-Sept 1959 N=337 All ages prospective population of Taiwan | JE (All but majority JE) | not specified | all but implied from paper majority assumed to be JE | not specified | HI test for JE CF test for JE | 6 |  |
| Chatterjee, et al (1975) [60] | **India (T), PY 1975** SY July-Oct 1973, N= 323, Prospective outbreak investigations, All ages | JE (All but majority JE) | nil specified | all but majority presumed to be JE implied from paper | Acute onset, fever and headache followed by altered mental state. | JE specific IgM ELISA | 159 |  |
| Stein-Zamir, et al. (2008) [61] | **Israel PY 2008** SY 2007 N=2 Prospective outbreak investigation All ages | Measles | measles only | measles encephalitis | not specified | IgM positive for measles or epidemiological link to another case | 0.23 (whole population )  0.74(< 16yrs) |  |
| **Reference** | **Setting Western/ Tropical (W/T); Study year SY; No of Cases (N=); Study type; Population under study** | **Aetiology** | **Exclusion Criteria** | **Aetiologies included** | **Clinical definition of AE case** | **Laboratory Diagnostic criteria for AE case** | **Annual Incidence**  ***(data converted to / 100 000 population)**  **incidence calculated from data in paper**** | **Notes** |
| Rey et al (1983) [62] | **France (W) PY 1983** SY 1977-1981 Epidemiological surveillance and retrospective hospital survey N=1 SSPE All ages | Measles and SSPE | not specified | Meales encephalitis and SSPE | not specified | not specified | 0.038 (SSPE) Measles encephalitis estimated at 1/ 2850 cases of measles |  |
| Ueda et al (1984) [63] | **Japan (W) PY 1984** SY 1975 to 1977 N=3, Retrospective questionnaire survey Japanese All ages | Rubella | Not specified | Rubella encephalitis only | not specified | not specified | 3.73** |  |
| Moriuchi, et al. (1990) [64] | **Japan (T) PY 1990** SY 1987 N= 5 Prospective Sasebo <15yrs | Rubella | Not specified | Rubella encephalitis only | not specified | Rising titre >= 4 fold or a high titre >= rubella HI antibody | 8.67** rubella encephalitis | in the context of a rubella epidemic |
| Ranzenhofer, et al. (1955) [65] | **Kentucky USA (W) PY 1957** SY 1956, N= 13 All ages Prospective outbreak investigation | SLE | non SLE encephalitis adults | SLE | not specified | acute and convalescent sera for complement fixation and in some patients neutralisation tests | 867 ** | in context of SLE epidemic |
| Altman, et al (1968), Goldfield, et al. (1968) [66, 67] | **Delaware, USA (W) PY 1968** SY 1964 N=58, Prospective outbreak investigation All ages | SLE |  | Confirmed SLE only | not specified | Viral isolation, HI , CF, Neutralisation tests for SLE antibody | 14 | in context of SLE epidemic |
| **Reference** | **Setting Western/ Tropical (W/T); Study year SY; No of Cases (N=); Study type; Population under study** | **Aetiology** | **Exclusion Criteria** | **Aetiologies included** | **Clinical definition of AE case** | **Laboratory Diagnostic criteria for AE case** | **Annual Incidence**  ***(data converted to / 100 000 population)**  **incidence calculated from data in paper**** | **Notes** |
| Hopkins, et al. (1975) [68] | **Dallas Texas USA (W), PY 1975** SY 1966 145 Confirmed SLE encephalitis N= 119 all encephalitis N= 182 , Prospective outbreak investigation All ages | SLE | SLE lab confirmed encephalitis clinical encephalitis only calculated from data in paper | all CNS patients hospitalized with CNS infections | serious febrile illness of unknown aetiology with severe headache and signs of CNS involvement predominating, eg tremor, ataxia, confusion, disorientation or alterations in state of consciousness | Sera initially screened using the HAI technique for activity against Group B antigen (SLE and Murray Valley encephalitis) Later CF tests on same sera. Positives were confirmed by assaying neutralizing antibodies. | 15.2 **(SLE)  19.2 (All reported encephalitis) | in context of SLE epidemic |
| Williams, (1975) [69] | **Texas USA (W) PY 1975** SY 1966 All ages N=60 Prospective outbreak investigation | SLE | SLE laboratory confirmed only | St Louis encephalitis | not specified | Haemagglutination inhibition (HAI) for SLE and Murray valley encephalitis CF, confirmation by assaying for neutralizing antibodies. | 41 (all SLE presentations )  32.0** (SLE encephalitis) | in context of SLE epidemic |
| Powell et al (1977) [70] | **Mississippi, USA (W), PY 1977**, SY 1975 N= 132, all ages. Prospective outbreak investigation | SLE | non SLE encephalitis | lab confirmed SLE | Fever, (temp >=37.8oC), neurologic abnormality of resent onset, Lethargy | Fourfold rise in HI or CF antibody titre agains SLE virus antigen or singel or multiple HI titres of 80 or greater if examined by CDC or titre of >=40 if only examined by State Lab. | 5.9** | in context of SLE epidemic |
| Maetz, et al. (1978) [71] | **Birmingham Alabama USA (W) 1978** SY 1975 n=20 encephalitis Prospective outbreak investigation all ages | SLE | only suspected cases of SLE reported | SLE confirmed encephalitis | not specified 20 of 45 clinical presentations with encephalitis confirmed as SLE encephalitis. | Serological testing for SLE, YF and EEE, WEE, VEE. Neutralisations tests as confirmation | 9.3** | attack rate greatest in 65-74 year age group |
| **Reference** | **Setting Western/ Tropical (W/T); Study year SY; No of Cases (N=); Study type; Population under study** | **Aetiology** | **Exclusion Criteria** | **Aetiologies included** | **Clinical definition of AE case** | **Laboratory Diagnostic criteria for AE case** | **Annual Incidence**  ***(data converted to / 100 000 population)**  **incidence calculated from data in paper**** | **Notes** |
| Marfin, et al. (1993) [72] | **Arkansas, USA PY 1993,** SY 1991; N=25 Prospective outbreak investigation All age groups | SLE | non-SLE encephalitis | SLE | Febrile illness (temperature >38oC) signs and symptoms of neuroinvasive disease such as headache, delirium, coma or new onset seizures | SLE virus specific IgM in CSF or serum or a fourfold or greater change in titre to SLE by hamagglutination antibody inhibition | 44 | in context of SLE epidemic |
| Luby,et al. (1967) [73] | **Houston Texas, USA (W) PY 1967** SY 1955-1971 Prospective outbreak investigation All ages | SLE | As used by Quick et al 1965 | St Louis encephalitis | clinical syndromes including: focal paralysis, disorientation, stupor, excessive drowsiness, tremor, ataxia and slurred speech also included aseptic meningitis, and headache and fever cases | HI for SLE, Murray Valley Encephalitis, WEE and EEE and neutralization tests CF introduced later | 19.5(overall)  8.2(0-9yrs),  108.8( >70yrs of whom 57.1% had encephalitis presentation) | in context of SLE epidemic |
| McGowan, et al. (1973) [74] | **USA (W) PY 1973** SY 1955-1971 N= 2206 Selected USA states Retrospective All ages | SLE | not specified | SLE serologically confirmed | not specified | methods, including serology, haemagglutination inhibition, complement fixation, suckling mouse neutralisation test | lowest 0.8 (age<0-/9 New Jersey ) to highest 309.8( Florida in >70 yrs) 62.2 (mean for listed states and all ages) |  |
| Gonzalez Cortes et al (1975) [75] | **Mexico (W) PY 1976** SY 1974 N= 51, Retrospective, All ages | SLE (All but majority SLE) | not specified | All but implied from paper majority assumed to be SLE | Encepahlomyelitis presentation: features included fever, vomiting, lethargy and neck stiffness | HI for SLE | 19 (Hospitalized cases with encephalitis) |  |
| **Reference** | **Setting Western/ Tropical (W/T); Study year SY; No of Cases (N=); Study type; Population under study** | **Aetiology** | **Exclusion Criteria** | **Aetiologies included** | **Clinical definition of AE case** | **Laboratory Diagnostic criteria for AE case** | **Annual Incidence**  ***(data converted to / 100 000 population)**  **incidence calculated from data in paper**** | **Notes** |
| Monath, (1979) [76] | **USA and Mexico (W) PY 1979** SY 1974-1977 N= 4824 SLE, Cal E 1035, WEE 947, EEE 136 Retrospective, All ages | SLE, Cal E, WEE, EEE | only diseases specified | SLE California encephalitis WEE Rocio VEE | not specified Probably includes all CNS arboviral presentations | not specified | 19 (SLE Mexico Hermosillo 1974 ) 1.2 to 151.3 (USA selected States 1974-77) |  |
| Campbell, et al. (2005) [77] | **Canada (W) PY 2005** SY 1997 - 2000 N=4, Prospective surveillance Population <19 years | SSPE | Canadian Paediatric Surveillance Program (CPSP | SSPE only | not specified |  | 0.6 |  |
| Mickiene, et al. (2002) [78] | **Lithuania (W); PY (2002)** SY 1997; N=250 prospective | TBE | TBE only only >= 16 years | TBE only bacterial and non-TBE encephalitis excluded. | all patients with clinical signs of neuroinfection and CSF cell count >= 8 x1o6cells/l | Specific IgM activity for TBE. CNS activity by demonstration of intrathecally produced TBE IgM antibodies. | 17.4 |  |
| Blaskovic (1967) [79] | **Tribec region of former Czechoslvakia (W) PY 1967** SY 1953-63 N= 458 Prospective All ages | TBE |  | Tick borne encephalitis | Meningo-encephalitis: biphasic disease but no specific details | serology complement fixing antibodies and virus neutralisation tests | 14.6 |  |
| **Reference** | **Setting Western/ Tropical (W/T); Study year SY; No of Cases (N=); Study type; Population under study** | **Aetiology** | **Exclusion Criteria** | **Aetiologies included** | **Clinical definition of AE case** | **Laboratory Diagnostic criteria for AE case** | **Annual Incidence**  ***(data converted to / 100 000 population)**  **incidence calculated from data in paper**** | **Notes** |
| Vutuc, (1994) [80] | **Austria (W) PY 199**4 SY 1990-1991 N=162 Retrospective, All ages | TBE |  | Hospitalised cases of TBE | (ICD 063, 0632) | not specified | 1.1 ( 1990)  0.88 (1991) |  |
| Kerbo, et al. (2005) [81] | **Estonia(W) PY 200**5 SY 1950-2004 Retrospective All population all ages | TBE | not specified | Tick borne encephalitis | Not specified | not specified | 0.1- 0.3 ( 1951 - 1975),  3 (1976-1992),  28 and 27 (1997 and 1998),  12 (1996),  13 (2004). | seasonal variation in incidence, |
| Blaskovic (1970) [82] | **Tribec region of former Czechoslvakia (W), PY 1970** (SY) 1953-67, N=14,553, Retrospective, All ages | TBE | Not specified | tick borne encephalitis Meningoencephalitis | not specified | Serologically confirmed TBE details not specified | 14.6 (mean over years 1953-1967) 5.2 **(Median range** (0-107.9)) |  |
| Suss (1992) [83] | **Germany (W) PY 1992,** SY 1960-1990 N=193 1(960-1985), N=11 (1986-1990) Retrospective, All ages | TBE | Not specified | All TBE presentations | not specified | TBE Sero-diagnosis, Neutralization test, CF and HI. | 0.7 (1960-1990),  0.2(Decreased since 1990) | Population of 5 Lander, |
| Pazdiora et al (2008) [84] | **Czech Republic (W) PY** 2008 SY 1960-2005 N=1621 Retrospective, All ages | TBE | Not specified | Lab confirmed TBE | not defined probably includes all presentations of TBE | Diagnostic tests varied over time, including CFreaction, HI test, virus neutralization test and ELISA. | 4.1,  21.7 (highest infection rate in district of Klatovy) |  |
| **Reference** | **Setting Western/ Tropical (W/T); Study year SY; No of Cases (N=); Study type; Population under study** | **Aetiology** | **Exclusion Criteria** | **Aetiologies included** | **Clinical definition of AE case** | **Laboratory Diagnostic criteria for AE case** | **Annual Incidence**  ***(data converted to / 100 000 population)**  **incidence calculated from data in paper**** | **Notes** |
| Schwanda, et al. (2000) [85] | **Switzerland (W) PY 2000,** (SY) 1990-1999; N= 73 Retrospective, All ages | TBE | Not specified | TBE only bacterial and non-TBE encephalitis excluded. | 32 (43.8% ) of cases presented with encephalitis, 24 (32.9%) with meningitis | Clinical syndrome plus TBE IgM in serum and CSF Western Blot was used in a few cases. | 2.2 (1990-95),  7.8 (1996-1999),  13.8 (1999) |  |
| Zenz, et al. (2005) [86] | **Austria and Slovenia (W) PY 2005** SY 1980-2003 N= 139 Austria, N=783 Slovenia Retrospective, Ages < 16 yrs | TBE | only TBE confirmed cases | TBE | TBE Meningitis and or meningo-encephalitis and investigation of VSF | Austria diagnosis by ELISA specific for TBE specific IgM and IgG antibodies in serum and or CSF since 1980 in Slovenia 1980 - 89 4 fold rise in antibody titre of complement fixation reaction. After 1990 detection of IgM and IgG antibodies against TBE | 2-5-9.3(1980-1986) 0-2-2 (1987 to 1993), 0-1(1994 to 2003) | TBE vaccination introduced in 1976 formalin inactivated whole virus vaccine |
| Stahelin-Massik et al. (2008) [87] | **Switzerland (W) PY 2008,** SY 2000-2004 N= 55 Prospective, <16yrs | TBE | Not specified | TBE only | TBE present of neurologic symptoms ( headache, vomiting , neck stiffness, altered states of consciousness and or flu like symptoms ) CSF > 10 leukocytes/ml and positive serology in blood or CSF | positive TBE serology detectable IgM antibodies, IgG seroconversion or a 4 fold rise in serum IgG antibodies | 1 (all children)  0.4 (children < 6 years) |  |
| Guess et al (1984) [88] | **Olmsted County USA (W) PY 1984** SY 1962-1981 N= 4 Retrospective survey all ages | VZV |  | varicella encephalitis | ICD -9CM (International Classification of Diseases 9th Revision, Clinical Modification) post varicella encephalitis 052.0 | not specified | 9 |  |
| Cameron, et al. (2007) [89] | **UK & Ireland (W) PY 2007** SY Nov 2002–Nov 2003. N=26 Prospective surveillance  Ages < 16 years | VZV | physician surveillance | encephalitis complicating varicella zoster | not specified | Laboratory diagnosis was most frequently serology, either alone (n=6) or with other tests (n=5), including PCR (n=2), culture (n=1), (n=1), or in one case, PCR, culture and immunofluorescence. Apart from serology, one case each was diagnosed by immunofluorescence alone, PCR alone, or a combination of PCR and electron microscopy or immunofluorescence and culture. | 0.19** | only children with VZV encephalitis |
| **Reference** | **Setting Western/ Tropical (W/T); Study year SY; No of Cases (N=); Study type; Population under study** | **Aetiology** | **Exclusion Criteria** | **Aetiologies included** | **Clinical definition of AE case** | **Laboratory Diagnostic criteria for AE case** | **Annual Incidence**  ***(data converted to / 100 000 population)**  **incidence calculated from data in paper**** | **Notes** |
| Huhn, et al. (2005) [90] | **Ilinois USA (W) PY 2005** SY 2002 N=311 Prospective outbreak investigation All ages | WNV | Not specified | West Nile encephalitis | not specified |  | 2.5** | in context of West Nile epidemic |
| Tsai, et al. (1998) [91] | **Romania (W) PY 1998** SY N= 393 West Nile Fever Prospective outbreak investigation All ages | WNV | WNV only | West Nile only | all CNS infections Encephalitis definition not specified but included disorientation and altered consciousness | serum and CSF for IgM and IgG antibodies to WN virus by capture and indirect EIA | 1.8** (encephalitis) 5.5** (meningo-encephalitis) 12.4 all WNV CNS infections) |  |
| Bode, et al. (2006) [92] | **Colorado USA (W) PY 2006**; SY 2003 N=65, Retrospective, All ages | WNV | only hospitalized West Nile cases |  | West Nile encephalitis if: fever ≥ 38oC, and at least one of the following characteristics, acutely altered mental status (ie disorientation, obtundation, stupor, coma or personality change the lasted > 24hours) neuroimaging findings consistent with acute cerebral inflammation or electroencephalgraphy findings consistent with encephalitis. | West Nile Virus specific IgM antibody in acute -phase serum of CSF | 7.4 |  |
| LaBeaud, et al. (2006) [93] | **Ohio USA (W) PY 2006** SY Aug 14-Dec 31 2002 N=5 Retrospective, Ages 5-17yrs | WNV | West Nile only neurologic admission | West Nile only | West Nile neurologic disease fever plus neurologic presentation including photophobia, meningism | serum or CSF positive for anti WNV IgM or IgG | 1.4 |  |

1. Kaeaeriaeinen L, Klemola E, Forssell P, Hirvonen E, Oker-Blom N: **[Acute Primary Encephalitis and Primary Serous (Aseptic) Meningitis in Finland.].** *Duodecim* 1964, **80:**361-373.

2. Klemola E, Kaeaeriaeinen L, Ollila O, Pettersson T, Jansson E, Haapanen L, Lapinleimu K, Forssell P: **Studies on viral encephalitis** *Acta Med Scand* 1965, **177:**707-716.

3. Beghi E, Nicolosi A, Kurland LT: **Encephalitis and aseptic meningitis, Olmsted County, Minnesota, 1950-1981: I. Epidemiology.** *Annals of Neurology* 1984, **16:**283-294.

4. Henrich TJ, Hutchaleelaha S, Jiwariyavej V, Barbazan P, Nitatpattana N, Yoksan S, Gonzalez J-P: **Geographic dynamics of viral encephalitis in Thailand.** *Microbes & Infection* 2003, **5:**603-611.

5. Ponka A, Pettersson T: **The incidence and aetiology of central nervous system infections in Helsinki in 1980.** *Acta Neurologica Scandinavica* 1982, **66:**529-535.

6. Nicolosi A, Hauser WA, Beghi E, Kurland LT: **Epidemiology of central nervous system infections in Olmsted County, Minnesota, 1950-1981.** *J Infect Dis* 1986, **154:**399-408.

7. Pedersen E: **Epidemic encephalitis in Jutland; a clinical survey for the years 1952-54.** *Dan Med Bull* 1956, **3:**65-75.

8. Khetsuriani N, Holman RC, Anderson LJ: **Burden of encephalitis-associated hospitalizations in the United States, 1988-1997.** *Clin Infect Dis* 2002, **35:**175-182.

9. Khetsuriani N, Holman RC, Lamonte-Fowlkes AC, Selik RM, Anderson LJ: **Trends in encephalitis-associated deaths in the United States.** *Epidemiol Infect* 2007, **135:**583-591.

10. Kamei S, Takasu T: **Nationwide survey of the annual prevalence of viral and other neurological infections in Japanese inpatients.** *Intern Med* 2000, **39:**894-900.

11. Trevejo RT: **Acute encephalitis hospitalizations, California, 1990-1999: unrecognized arboviral encephalitis?** *Emerg Infect Dis* 2004, **10:**1442-1449.

12. Laursen K, Knudsen JD: **Tick-borne encephalitis: A retrospective study of clinical cases in Bornholm, Denmark.** *Scandinavian Journal of Infectious Diseases* 2003, **35:**354-357.

13. Mailles A, Vaillant V, Stahl JP: **[Infectious encephalitis in France from 2000 to 2002: the hospital database is a valuable but limited source of information for epidemiological studies].** *Médecine et maladies infectieuses* 2007, **37:**95-102.

14. Kupila L, Vuorinen T, Vainionpaa R, Hukkanen V, Marttila RJ, Kotilainen P: **Etiology of aseptic meningitis and encephalitis in an adult population.** *Neurology* 2006, **66:**75-80.

15. Radhakrishnan K, Maloo JC, Poddar SK, Mousa ME: **Central nervous system infections in Benghazi, Libya: experience from a community-based adult medical neurology set-up.** *Journal of Tropical Medicine & Hygiene* 1987, **90:**123-126.

16. Nwosu CM, Njeze GE, Opara C, Nwajuaku C, Chukwurah CK: **Central nervous system infections in the rainforest zone of Nigeria.** *East African Medical Journal* 2001, **78:**97-101.

17. Rantalaiho T, Farkkila M, Vaheri A, Koskiniemi M: **Acute encephalitis from 1967 to 1991.** *J Neurol Sci* 2001, **184:**169-177.

18. Davison KL, Crowcroft NS, Ramsay ME, Brown DW, Andrews NJ: **Viral encephalitis in England, 1989-1998: what did we miss?** *Emerg Infect Dis* 2003, **9:**234-240.

19. Rantakallio P, Leskinen M, von Wendt L: **Incidence and prognosis of central nervous system infections in a birth cohort of 12,000 children.** *Scandinavian Journal of Infectious Diseases* 1986, **18:**287-294.

20. Wang D, Bortolussi R: **Acute viral infection of the central nervous system in children: an 8-year review.** *Can Med Assoc J* 1981, **125:**585-589.

21. Wong V, Yeung CY: **Acute viral encephalitis in children.** *Aust Paediatr J* 1987, **23:**339-342.

22. Ilias A, Galanakis E, Raissaki M, Kalmanti M: **Childhood encephalitis in Crete, Greece.** *J Child Neurol* 2006, **21:**910-912.

23. Ishikawa T, Asano Y, Morishima T, Nagashima M, Sobue G, Watanabe K, Yamaguchi H: **Epidemiology of acute childhood encephalitis. Aichi Prefecture, Japan, 1984-90.** *Brain Dev* 1993, **15:**192-197.

24. Rantala H, Uhari M: **Occurrence of childhood encephalitis: a population-based study.** *Pediatr Infect Dis J* 1989, **8:**426-430.

25. Kolski H, Ford-Jones EL, Richardson S, Petric M, Nelson S, Jamieson F, Blaser S, Gold R, Otsubo H, Heurter H, MacGregor D: **Etiology of acute childhood encephalitis at The Hospital for Sick Children, Toronto, 1994-1995.** *Clin Infect Dis* 1998, **26:**398-409.

26. Cizman M, Jazbec J: **Etiology of acute encephalitis in childhood in Slovenia.** *Pediatr Infect Dis J* 1993, **12:**903-908.

27. Koskiniemi M, Vaheri A: **Effect of measles, mumps, rubella vaccination on pattern of encephalitis in children.** *Lancet* 1989, **1:**31-34.

28. Koskiniemi M, Korppi M, Mustonen K, Rantala H, Muttilainen M, Herrgard E, Ukkonen P, Vaheri A: **Epidemiology of encephalitis in children. A prospective multicentre study.** *Eur J Pediatr* 1997, **156:**541-545.

29. Bond JO: **St. Louis encephalitis and dengue fever in the Caribbean area: evidence of possible cross-protection.** *Bull World Health Organ* 1969, **40:**160-163.

30. Quick DT, Thompson JM, Bond JO: **The 1962 Epidemic of St. Louis Encephalitis in Florida. Iv. Clinical Features of Cases Occurring in the Tampa Bay Area.** *American Journal of Epidemiology* 1965, **81:**415-427.

31. Leake JP: **Epidemiology of Encephalitis: With Special Reference to the 1933 Epidemic.** *Am J Public Health Nations Health* 1933, **23:**1140-1143.

32. Chakrabarty S, Saxena VK, Bhardwaj M: **Epidemiological investigations of Japanese encephalitis outbreak in Gorakhpur and Deoria districts of Uttar Pradesh 1985.** *J Commun Dis* 1986, **18:**103-108.

33. Yamada T, Rojanasuphot S, Takagi M, Wungkobkiat S, Hirota T: **Studies on an epidemic of Japanese encephalitis in the northern region of Thailand in 1969 and 1970.** *Biken J* 1971, **14:**267-296.

34. Vajpayee A, Dey PN, Chakraborty AK, Chakraborty MS: **Study of the outbreak of Japanese encephalitis in Lakhimpur district of Assam in 1989.** *J Indian Med Assoc* 1992, **90:**114-115.

35. Narasimham MV, Rao CK, Bendle MS, Yadava RL, Johri YC, Pandey RS: **Epidemiological investigation on Japanese encephalitis outbreak in Uttar Pradesh during 1988.** *J Commun Dis* 1988, **20:**263-275.

36. Kar NJ, Saxena VK: **Some epidemiological characteristics of Japanese encephalitis in Haryana state of northern India.** *J Commun Dis* 1998, **30:**129-131.

37. Rubin RH, Glick TH, Rose NJ: **St. Louis encephalitis in Saline County, Illinois, 1968.** *J Infect Dis* 1970, **122:**347-353.

38. Najioullah F, Bosshard S, Thouvenot D, Boibieux A, Menager B, Biron F, Aymard M, Lina B: **Diagnosis and surveillance of herpes simplex virus infection of the central nervous system.** *Journal of Medical Virology* 2000, **61:**468-473.

39. Skoldenberg B, Forsgren M, Alestig K, Bergstrom T, Burman L, Dahlqvist E, Forkman A, Fryden A, Lovgren K, Norlin K, et al.: **Acyclovir versus vidarabine in herpes simplex encephalitis. Randomised multicentre study in consecutive Swedish patients.** *Lancet* 1984, **2:**707-711.

40. Hjalmarsson A, Blomqvist P, Skoldenberg B: **Herpes simplex encephalitis in Sweden, 1990-2001: incidence, morbidity, and mortality.** *Clin Infect Dis* 2007, **45:**875-880.

41. Omland LH, Vestergaard BF, Wandall JH: **Herpes simplex virus type 2 infections of the central nervous system: A retrospective study of 49 patients.** *Scandinavian Journal of Infectious Diseases* 2008, **40:**59-62.

42. Grossman RA, Edelman R, Chiewanich P, Voodhikul P, Siriwan C: **Study of Japanese encephalitis virus in Chiangmai valley, Thailand. II. Human clinical infections.** *Am J Epidemiol* 1973, **98:**121-132.

43. Okuno T, Tseng PT, Hsu ST, Huang CT, Kuo CC: **Japanese encephalitis surveillance in China (Province of Taiwan) during 1968-1971. II. Age-secific incidence in connection with Japanese encephalitis vaccination program.** *Jpn J Med Sci Biol* 1975, **28:**255-267.

44. Okuno T, Tseng PT, Hsu ST, Huang CT, Kuo CC: **Japanese encephalitis surveillance in China (Province of Taiwan) during 1968-1971. I. Geographical and seasonal features of case outbreaks.** *Jpn J Med Sci Biol* 1975, **28:**235-253.

45. Wu YC, Huang YS, Chien LJ, Lin TL, Yueh YY, Tseng WL, Chang KJ, Wang GR: **The epidemiology of Japanese encephalitis on Taiwan during 1966-1997.** *Am J Trop Med Hyg* 1999, **61:**78-84.

46. Chunsuttiwat S: **Japanese encephalitis in Thailand.** *Southeast Asian Journal of Tropical Medicine & Public Health* 1989, **20:**593-597.

47. Wang LH, Fu SH, Wang HY, Liang XF, Cheng JX, Jing HM, Cai GL, Li XW, Ze WY, Lv XJ, et al: **Japanese encephalitis outbreak, Yuncheng, China, 2006.** *Emerg Infect Dis* 2007, **13:**1123-1125.

48. Hsieh WC, Wang SP, Rasmussen AF: **Epidemiology of Japanese encephalitis (JE) on Taiwan in 1960.** *Journal of the Formosan Medical Association* 1961, **60:**825-830.

49. Partridge J, Ghimire P, Sedai T, Bista MB, Banerjee M: **Endemic Japanese encephalitis in the Kathmandu valley, Nepal.** *Am J Trop Med Hyg* 2007, **77:**1146-1149.

50. Akiba T, Osaka K, Tang S, Nakayama M, Yamamoto A, Kurane I, Okabe N, Umenai T: **Analysis of Japanese encephalitis epidemic in Western Nepal in 1997.** *Epidemiol Infect* 2001, **126:**81-88.

51. Thongcharoen P: **Japanese encephalitis in Thailand.** *Journal of the Medical Association of Thailand* 1985, **68:**534-545.

52. Hashimoto S, Kawado M, Murakami Y, Izumida M, Ohta A, Tada Y, Shigematsu M, Yasui Y, Taniguchi K, Nagai M: **Epidemics of vector-borne diseases observed in infectious disease surveillance in Japan, 2000-2005.** *J Epidemiol* 2007, **17 Suppl:**S48-55.

53. Paul WS, Moore PS, Karabatsos N, Flood SP, Yamada S, Jackson T, Tsai TF: **Outbreak of Japanese encephalitis on the island of Saipan, 1990.** *J Infect Dis* 1993, **167:**1053-1058.

54. Kari K, Liu W, Gautama K, Mammen MP, Jr., Clemens JD, Nisalak A, Subrata K, Kim HK, Xu ZY: **A hospital-based surveillance for Japanese encephalitis in Bali, Indonesia.** *BMCMed* 2006, **4:**8.

55. Hoke CH, Nisalak A, Sangawhipa N, Jatanasen S, Laorakapongse T, Innis BL, Kotchasenee S, Gingrich JB, Latendresse J, Fukai K: **Protection against Japanese encephalitis by inactivated vaccines.** *NEnglJ Med* 1988, **319:**608-614.

56. Vijayarani H, Gajanana A: **Low rate of Japanese encephalitis infection in rural children in Thanjavur district (Tamil Nadu), an area with extensive paddy cultivation.** *Indian Journal of Medical Research* 2000, **111:**212-214.

57. Gajanana A, Thenmozhi V, Samuel PP, Reuben R: **A community-based study of subclinical flavivirus infections in children in an area of Tamil Nadu, India, where Japanese encephalitis is endemic.** *BullWorld Health Organ* 1995, **73:**237-244.

58. Tigertt WD, Berge TO: **Japanese B encephalitis.** *Am J Public Health Nations Health* 1957, **47:**713-718; contd.

59. Grayston JT, Wang SP, Yen CH: **Encephalitis on Taiwan. I. Introduction and epidemiology.** *American Journal of Tropical Medicine & Hygiene* 1962, **11:**126-130.

60. Chatterjee AK, Banerjee K: **Epidemiological studies on the encephalitis epidemic in Bankura.** *Indian J Med Res* 1975, **63:**1164-1179.

61. Stein-Zamir C, Abramson N, Shoob H, Zentner G: **An outbreak of measles in an ultra-orthodox Jewish community in Jerusalem, Israel, 2007--an in-depth report.** 2008**:**Bulletin Europeen sur les Maladies Transmissibles = European Communicable Disease Bulletin. 13(18), 2008 Feb 2021.

62. Rey M, Celers J, Mouton Y, Netter R: **Impact of measles in France.** *Reviews of Infectious Diseases* 1983, **5:**433-438.

63. Ueda K, Sasaki F, Tokugawa K, Segawa K, Fujii H: **The 1976-1977 rubella epidemic in Fukuoka city in southern Japan: epidemiology and incidences of complications among 80,000 persons who were school children at 28 primary schools and their family members.** *Biken Journal* 1984, **27:**161-168.

64. Moriuchi H, Yamasaki S, Mori K, Sakai M, Tsuji Y: **A rubella epidemic in Sasebo, Japan in 1987, with various complications.** *Acta Paediatrica Japonica* 1990, **32:**67-75.

65. Ranzenhofer ER, Alexander ER, Beadle LD, Bernstein A, Pickard RC: **St. Louis encephalitis in Calvert City, Kentucky, 1955; an epidemiologic study.** *Am J Hyg* 1957, **65:**147-161.

66. Goldfield M, Altman R, Welsh JN, Taylor BF, Pizzuti W, Black HC, Mazzur SR, Bill JS: **The 1964 outbreak of St. Louis encephalitis in the Delaware Valey. 2. Laboratory studies of cases and serologic diagnostic procedures.** *Am J Epidemiol* 1968, **87:**470-483.

67. Altman R, Goldfield M: **The 1964 outbreak of St. Louis encephalitis in the Delaware Valley 1. Description of outbreak.** *Am J Epidemiol* 1968, **87:**457-469.

68. Hopkins CC, Hollinger FB, Johnson RF, Dewlett HJ, Newhouse VF, Chamberlain RW: **The epidemiology of St. Louis encephalitis in Dallas, Texas, 1966.** *Am J Epidemiol* 1975, **102:**1-15.

69. Williams KH, Hollinger FB, Metzger WR, Hopkins CC, Chamberlain RW: **The epidemiology of St. Louis encephalitis in Corpus Christi, Texas, 1966.** *Am J Epidemiol* 1975, **102:**16-24.

70. Powell KE, Blakey DL: **St Louis encephalitis. The 1975 epidemic in Mississippi.** *JAMA* 1977, **237:**2294-2298.

71. Maetz HM, Pate P, Sellers C, Bailey WC, Holmes R, Hardy GE, Jr.: **Epidemiology and control of St. Louis encephalitis in Birmingham, Alabama, 1975.** *Am J Public Health* 1978, **68:**588-590.

72. Marfin AA, Bleed DM, Lofgren JP, Olin AC, Savage HM, Smith GC, Moore PS, Karabatsos N, Tsai TF: **Epidemiologic aspects of a St. Louis encephalitis epidemic in Jefferson County Arkansas, 1991.** *Am J Trop Med Hyg* 1993, **49:**30-37.

73. Luby JP, Miller G, Gardner P, Pigford CA, Henderson BE, Eddins D: **The epidemiology of St. Louis encephalitis in Houston, Texas, 1964.** *Am J Epidemiol* 1967, **86:**584-597.

74. McGowan JE, Jr., Bryan JA, Gregg MB: **Surveillance of arboviral encephalitis in the United States, 1955-1971.** *Am J Epidemiol* 1973, **97:**199-207.

75. Gonzalez Cortes A, Zarate Aquino ML, Guzman Bahena J, Miro Abella J, Cano Avila G, Aguilera Arrayo M: **St. Louis encephalomyelitis in Hermosillo, Sonora, Mexico.** *Bull Pan Am Health Organ* 1975, **9:**306-316.

76. Monath TP: **Arthropod-borne encephalitides in the Americas.** *Bull World Health Organ* 1979, **57:**513-533.

77. Campbell C, Levin S, Humphreys P, Walop W, Brannan R: **Subacute sclerosing panencephalitis: results of the Canadian Paediatric Surveillance Program and review of the literature.** *BMC Pediatr* 2005, **5:**47.

78. Mickiene A, Laiskonis A, Gunther G, Vene S, Lundkvist A, Lindquist L: **Tickborne encephalitis in an area of high endemicity in lithuania: disease severity and long-term prognosis.** *Clin Infect Dis* 2002, **35:**650-658.

79. Blaskovic D, Pucekova G, Kubinyi L, Stupalova S, Oravcova V: **An epidemiological study of tick-borne encephalitis in the Tribec region: 1953-63.** *Bull World Health Organ* 1967, **36:**Suppl 1:89-94.

80. Vutuc C, Kunze M: **Tick-borne encephalitis in Austria: incidence 1990 and 1991.** *Eur J Epidemiol* 1994, **10:**343-344.

81. Kerbo N, Donchenko I, Kutsar K, Vasilenko V: **Tickborne encephalitis epidemiology in Estonia, 1950-2004.** *Euro Surveill* 2005, **10:**E050630 050637.

82. Blaskovic D: **Tick-borne encephalitis in Czechoslovakia.** *Arch Environ Health* 1970, **21:**453-461.

83. Suss J: **Epidemiology and ecology of TBE relevant to the production of effective vaccines.** *Vaccine* 2003, **21:**S19-S35.

84. Pazdiora P, Benesova J, Bohmova Z, Kralikova J, Kubatova A, Menclova I, Moravkova I, Pruchova J, Prechova M, Spacilova M, et al: **The prevalence of tick-borne encephalitis in the region of West Bohemia (Czech Republic) between 1960-2005.** *Wiener Medizinische Wochenschrift* 2008, **158:**91-97.

85. Schwanda M, Oertli S, Frauchiger B, Krause M: **[Tick-borne meningoencephalitis in Thurgau Canton: a clinical and epidiomological analysis].** *Schweizerische Medizinische Wochenschrift* 2000, **Journal Suisse de Medecine. 130:**1447-1455.

86. Zenz W, Pansi H, Zoehrer B, Mutz I, Holzmann H, Kraigher A, Berghold A, Spork D: **Tick-borne encephalitis in children in Styria and Slovenia between 1980 and 2003.** *Pediatr Infect Dis J* 2005, **24:**892-896.

87. Stahelin-Massik J, Zimmermann H, Gnehm HE: **Tick-borne encephalitis in Swiss children 2000-2004: five-year nationwide surveillance of epidemiologic characteristics and clinical course.** *Pediatr Infect Dis J* 2008, **27:**555-557.

88. Guess HA, Broughton DD, Melton LJ, 3rd, Kurland LT: **Chickenpox hospitalizations among residents of Olmsted County, Minnesota, 1962 through 1981. A population-based study.** *American Journal of Diseases of Children* 1984, **138:**1055-1057.

89. Cameron JC, Allan G, Johnston F, Finn A, Heath PT, Booy R: **Severe complications of chickenpox in hospitalised children in the UK and Ireland.[see comment].** *Archives of Disease in Childhood* 2007, **92:**1062-1066.

90. Huhn GD, Austin C, Langkop C, Kelly K, Lucht R, Lampman R, Novak R, Haramis L, Boker R, Smith S, et al: **The emergence of west nile virus during a large outbreak in Illinois in 2002.** *American Journal of Tropical Medicine & Hygiene* 2005, **72:**768-776.

91. Tsai TF, Popovici F, Cernescu C, Campbell GL, Nedelcu NI: **West Nile encephalitis epidemic in southeastern Romania.** *Lancet* 1998, **352:**767-771.

92. Bode AV, Sejvar JJ, Pape WJ, Campbell GL, Marfin AA: **West Nile Virus disease: A descriptive study of 228 patients hospitalized in a 4-county region of Colorado in 2003.** *Clinical Infectious Diseases* 2006, **42:**1234-1240.

93. LaBeaud AD, Lisgaris MV, King CH, Mandalakas AM: **Pediatric West Nile virus infection: neurologic disease presentations during the 2002 epidemic in Cuyahoga County, Ohio.** *Pediatr Infect Dis J* 2006, **25:**751-753.
